# Chromatin variation associated with liver metabolism is mediated by transposable elements

**DOI:** 10.1186/s13072-016-0078-0

**Published:** 2016-07-08

**Authors:** Juan Du, Amy Leung, Candi Trac, Michael Lee, Brian W. Parks, Aldons J. Lusis, Rama Natarajan, Dustin E. Schones

**Affiliations:** Department of Diabetes Complications and Metabolism, Beckman Research Institute, City of Hope, Duarte, CA USA; Irell & Manella Graduate School of Biological Sciences, City of Hope, Duarte, CA USA; Department of Nutritional Sciences, University of Wisconsin-Madison, Madison, WI USA; Department of Medicine, University of California, Los Angeles, CA USA

**Keywords:** Chromatin accessibility, Transposable element, Transcription factor, DNA methylation, FAIRE-seq

## Abstract

**Background:**

Functional regulatory regions in eukaryotic genomes are characterized by the disruption of nucleosomes leading to accessible chromatin. The modulation of chromatin accessibility is one of the key mediators of transcriptional regulation, and variation in chromatin accessibility across individuals has been linked to complex traits and disease susceptibility. While mechanisms responsible for chromatin variation across individuals have been investigated, the overwhelming majority of chromatin variation remains unexplained. Furthermore, the processes through which the variation of chromatin accessibility contributes to phenotypic diversity remain poorly understood.

**Results:**

We profiled chromatin accessibility in liver from seven strains of mice with phenotypic diversity in response to a high-fat/high-sucrose (HF/HS) diet and identified reproducible chromatin variation across the individuals. We found that sites of variable chromatin accessibility were more likely to coincide with particular classes of transposable elements (TEs) than sites with common chromatin signatures. Evolutionarily younger long interspersed nuclear elements (LINEs) are particularly likely to harbor variable chromatin sites. These younger LINEs are enriched for binding sites of immune-associated transcription factors, whereas older LINEs are enriched for liver-specific transcription factors. Genomic region enrichment analysis indicates that variable chromatin sites at TEs may function to regulate liver metabolic pathways. CRISPR-Cas9 deletion of a number of variable chromatin sites at TEs altered expression of nearby metabolic genes. Finally, we show that polymorphism of TEs and differential DNA methylation at TEs can both influence chromatin variation.

**Conclusions:**

Our results demonstrate that specific classes of TEs show variable chromatin accessibility across strains of mice that display phenotypic diversity in response to a HF/HS diet. These results indicate that chromatin variation at TEs is an important contributor to phenotypic variation among populations.

**Electronic supplementary material:**

The online version of this article (doi:10.1186/s13072-016-0078-0) contains supplementary material, which is available to authorized users.

## Background

Accessible (open) chromatin is a common feature of active regulatory regions in eukaryotic genomes [1, 2]. The cell type-specific accessibility of chromatin allows regulatory factors to bind to the underlying DNA, leading to tightly regulated gene expression [1, 3, 4]. Accessible chromatin regions have been shown to be variable among different individuals [1, 5, 6], and these variable chromatin sites have been shown to be associated with complex traits and disease susceptibility [7]. However, the mechanisms underlying chromatin accessibility variation, and the processes through which this variation impacts phenotypic diversity, remain poorly understood.

Initial investigations into the relationship between variation of chromatin accessibility and genetic variation have begun to elucidate some principles. Examination of chromatin signatures in individuals with diverse ancestries revealed extensive variation in regulatory regions and evidence of heritability of these signatures [6]. Chromatin accessibility profiling in human lymphoblastoid cell lines revealed the association of chromatin accessibility signatures with genetic variants which are associated with the expression of nearby genes and potentially phenotypic diversity in humans [5, 8]. A study in erythroblasts from eight strains of inbred mice found that approximately 1/3 of variable open chromatin sites can be explained by single nucleotide variants and that these variants were associated with complex traits and disease [7]. While these pioneering studies have provided some insight into the drivers of chromatin variation, the majority of chromatin variation across the genome remains unexplained.

In addition to single nucleotide variants, transposable elements (TEs) constitute a major portion of genomic variation [9, 10]. Approximately 50 % of the human genome and 40 % of the mouse genome are derived from TEs [11, 12]. TEs can affect nearby gene activity and have been linked to complex traits and diseases, including cancer and diabetes [13, 14]. Due to the deleterious nature of TE transposition, mammalian systems have a number of transcriptional and posttranscriptional mechanisms to silence TEs [15]. The major mechanisms responsible for the suppression of TE transposition are DNA methylation, histone methylation and RNA interference [15–17]. Most DNA methylation in mammals occurs within TE sequences in order to transcriptionally suppress TE activities [17, 18]. Indeed, in somatic cells, most TEs are epigenetically silenced by DNA methylation [19]. However, studies have shown that specific TEs can be derepressed in a tissue-specific manner [19–21]. For example, tissue-specific DNA hypomethylation within TEs has been shown to contribute to novel regulatory networks [19].

There is growing evidence that TEs have evolved for the benefit of the host, contributing to host genome expansion and genetic innovation [22]. TEs can regulate gene expression by functioning as distal enhancers, alternative promoters or alternative splicing signals [19, 20, 23, 24]. Chromatin accessibility at TEs has been associated with the transcription of nearby genes in a tissue-specific manner [25, 26]. Many binding sites for transcription factors (TFs) have been characterized within specific TE sequences [26, 27]. Analysis of TE-associated TF binding sites in different species has further suggested that the expansion of the mammalian TF binding repertoire has been mediated by TE transposition [24, 27]. Given the prevalence of TE sequences and their potential regulatory functions, we hypothesized that TEs can play a regulatory role in mouse liver, and the chromatin accessibility variation at TEs among different individual may drive phenotypic diversity among them.

To study the roles of TEs in chromatin accessibility variation, we chose seven strains of inbred mice that have differential response to a “western” high-fat, high-sucrose (HF/HS) diet [28] and performed genome-wide chromatin accessibility profiling in liver tissue using FAIRE-seq [29]. Given that TEs are typically repressed in somatic cells [15, 17], we expected that most TE sequences would be less accessible in mouse liver. Interestingly, we found that a substantial fraction of variable chromatin sites are at TEs. Furthermore, TE-associated region of chromatin variations among different strains regulates nearby metabolic genes. Taken together, our study shows that TE loci are sources of chromatin accessibility variation and metabolic gene regulation among different inbred strains, which may further impact phenotypic diversity in livers of different strains of mice.

## Results

### Chromatin accessibility variation observed in livers of mice with differential phenotypes

Previous studies have reported strain-specific heterogeneity in physiological response to HF/HS diet feeding [28, 30]. In this study, we chose male mice from seven commonly used inbred strains of mice: A/J, AKR/J, BALB/cJ, C57BL/6J, C3H/HeJ, CBA/J and DBA/2J. These mice display diverse body fat percentage change after 8 weeks of HF/HS feeding, ranging from an average increase of 70 % (BALB/cJ) to over 200 % (C57BL/6J) [28]. We also observed significant variation of liver phenotypic markers, including liver triglyceride content (Additional file 1: Figure S1) [31], as expected given the important metabolic functions of the liver [32].

**Fig. 1:**
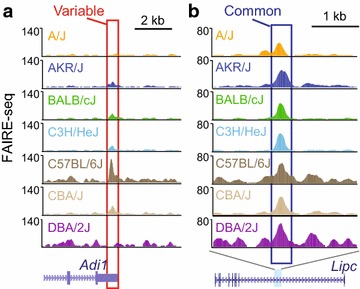
Chromatin accessibility across inbred strains of mice. Genome browser view of FAIRE-seq tracks for seven strains and RefSeq genes illustrating **a** a variable chromatin site (*boxed* in *red*) at the *Adi1* locus and **b** a common chromatin site (*boxed* in *blue*) at the *Lipc* locus

To profile chromatin accessibility at a genome-wide level, we performed FAIRE-seq [29] in livers from male mice of the seven strains after 8 weeks of HF/HS feeding (two biological replicates for each strain). In order to mitigate alignment biases, we created strain-specific pseudo-genomes using known single nucleotide polymorphisms (SNPs) [33], as described previously, and mapped the reads for each strain to the corresponding pseudo-genome [7]. With the aligned reads, we utilized F-seq [34] with the IDR framework [35] to identify reproducible peaks from our FAIRE-seq data for each of the seven strains. Using this approach, on average 29,752 reproducible accessible chromatin sites were identified in each individual strain (Additional file 1: Table S1). Combining the sites from the seven strains, we found a union set of 50,775 open chromatin sites. To identify sites that display variation in chromatin accessibility among the strains, we compared quantile-normalized read counts at the union set of sites using the DESeq package [7, 36]. We ranked sites by their adjusted *p* values (Additional file 1: Figure S2a) and selected the top 5 % as the most variable set of sites (2539 sites; adjusted *p* < 1.21e-9). Similarly, we classified the bottom 5 % of sites as the common set of sites. Variable sites display substantial heterogeneity in patterns of chromatin accessibility across the strains (Additional file 1: Figure S2b), indicating that the observed variability is not due to one strain being dramatically different from the others. Examples of variable and common chromatin sites are shown in Fig. 1.

Given that SNPs have been shown to contribute to chromatin variation in mouse erythroblasts [7], we first tested whether SNPs are associated with chromatin variation in the liver (see “Methods” section), and found that 30 % (764/2539) of the most variable chromatin sites have underlying SNPs that are associated with chromatin variation among the seven strains (Additional file 1: Figure S3a, b). This result is consistent with a previous study using erythroblasts from eight strains of inbred mice [7]. While this analysis provides a genetic explanation for ~1/3 of chromatin variation, the majority of chromatin variation among the inbred strains remained unexplained.

### Chromatin variability at TEs across inbred strains

Previous studies have shown that TEs contribute to regulatory networks in mammalian genomes [26]. We therefore reasoned that TEs could influence chromatin accessibility variation among inbred strains. Given that TEs are typically repressed/silenced in somatic cells [15, 17], we expected that TEs would be less enriched at sites of chromatin accessibility compared with random sites. To test this, we examined the prevalence of TEs in all accessible chromatin sites utilizing the RepeatMasker [37] annotation of TEs. As expected, sites of accessible chromatin are less likely to overlap instances of four classes of TEs (DNA transposons and the retrotransposon classes of LINEs (long interspersed nuclear elements), SINEs (short interspersed nuclear elements) and LTRs (long terminal repeats)) compared to random sites in the genome (34 vs. 54 %, *p* < 2.2 × e^−16^, Fisher’s exact test; Additional file 1: Figure S4a, b). These percentages are comparable to a previous study using DNase I hypersensitivity data sets from human tissues [26].

**Fig. 2:**
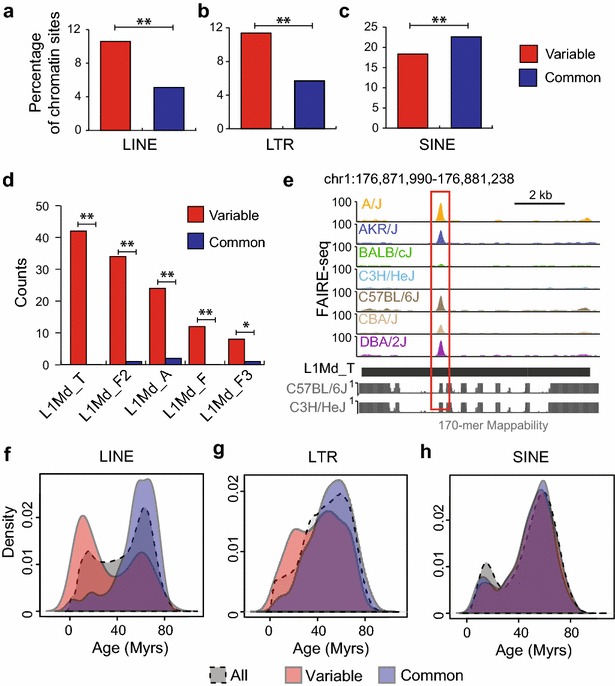
Specific subfamilies of TEs are enriched for variable chromatin sites. **a**–**c** Percentage of variable and common chromatin sites overlapping **a** LINEs, **b** LTRs and **c** or SINEs (**indicates *p* < 0.001, Fisher’s exact test). **d** Numbers of variable (*red*) or common (*blue*) chromatin sites overlapping certain subfamilies of TEs (*p < 0.05, **p < 0.001, Fisher’s exact test). **e** Genome browser view of a variable chromatin site in an L1Md_T element. The 170-mer mappability tracks for the reference genome and one representative pseudo-genome are shown below. **f**–**h** Age distribution of all TEs and TEs at variable or common chromatin sites for different classes of TEs, including **f** LINEs, **g** LTRs and **h** SINEs. *Myrs* million years

Interestingly, although TE sequences generally display less accessible chromatin, there are more TEs observed at variable chromatin sites than at common chromatin sites (37 vs 32 %, *p* = 0.001, Fisher’s exact test; Additional file 1: Figure S4c, d). Furthermore, two specific classes of retrotransposons, LINEs and LTRs, are significantly more enriched at variable chromatin sites compared with common chromatin sites (Fig. 2a, b; LINE: *p* = 3.7 × e^−13^; LTR: *p* = 4.5 × e^−13^, Fisher’s exact test). As an example, the variable chromatin site at the *Adi1* locus in Fig. 1a coincides with a LTR (Additional file 1: Figure S4e). In contrast, SINEs are more enriched at common chromatin sites compared with variable sites (Fig. 2c, *p* = 0.00017, Fisher’s exact test). DNA transposons are not enriched at either variable or common sites (Additional file 1: Figure S5a, *p* = 0.37, Fisher’s exact test).

### Variable chromatin sites are enriched at evolutionarily younger LINEs

Since specific subfamilies of TEs can play specific role in gene regulation [27, 38], we next investigated whether variable chromatin sites are enriched for specific families of TEs. Similar to previous analysis [24], we used the RepeatMasker [37] annotation of TE families and subfamilies and tabulated the occurrences of TEs from each subfamily at variable or common chromatin sites (Additional file 2: Table S2). Intriguingly, we found that several L1Md subfamilies are significantly enriched at the variable sites compared with common sites (Fig. 2d, e). These L1Md subfamilies of TEs are evolutionarily younger compared with other TEs with the average age of L1Md_T, L1Md_F2, L1Md_A, L1Md_F and L1Md_F3 being 8.27, 15.06, 8.05, 30.29 and 12.05 million years, respectively (see “Methods” section) [27, 37]. Furthermore, the accessibility of chromatin at younger L1Md subfamilies seems to be strain specific, where strains with higher chromatin accessibility at one young L1Md subfamily also showed higher accessibility for other young L1Md subfamilies (Additional file 1: Figure S6). Given that evolutionarily younger LINEs have diverged less and therefore contain less unique sequence, we assessed the potential of mapping biases by generating mappability tracks for the reference genome and representative pseudo-genomes for 170-mers, the average length of our mapped fragments (see “Methods” section).

To begin to access the potential association between genotype and strain-specific accessibility at young L1Mds, we profiled chromatin accessibility from two recombinant inbred strains, BXH2/TyJ and BXH19/TyJ, derived from C57BL/6J and C3H/HeJ. C57BL/6J has higher accessibility at younger L1Mds than does C3H/HeJ (Additional file 1: Figure S6). Interestingly, BXH2/TyJ has similar accessibility at young L1Mds compared with that of C57BL/6J, while BXH19/TyJ is more similar to C3H/HeJ (Additional file 1: Figure S7a). We further assessed whether the L1Mds that are commonly accessible in C57BL/6J and BXH2/TyJ but not C3H/HeJ and BXH19/TyJ can be explained by local genetic variants. We found 35 % (14/40) of these L1Mds are regions where C57BL/6J and BXH2/TyJ share a genotype at the locus, while C3H/HeJ and BXH19/TyJ share a different genotype (Additional file 1: Supplementary methods). Given the known roles of suppressor proteins and epigenetic modifications in controlling chromatin accessibility [15], it is not surprising that local genetic variation does not explain all of chromatin variation. Nevertheless, we did find examples where accessibility of LINE corresponds to the genotype (Additional file 1: Figure S7b).

Given that sites of variable chromatin are enriched for evolutionarily younger families of LINE elements compared with common sites, we next asked whether TEs at variable sites are in general evolutionarily younger than those at common sites. We again separated TEs into four classes (DNA transposons and SINE, LINE and LTR retrotransposons) and plotted the distribution of the evolutionary age of all elements as well as those at variable and common sites separately in each of the classes (Fig. 2f–h; Additional file 1: Figure S5b). Strikingly, we found that LINEs at variable chromatin sites display a bimodal distribution for age, with one subgroup of evolutionarily younger LINEs being prominently variable (Fig. 2f). In contrast, LINEs that overlap common chromatin sites are in general evolutionarily older (Fig. 2f, *p* < 2.2 × e^−16^). This difference between variable and common sites was further exemplified when we grouped individual LINEs into subfamilies (Additional file 1: Figure S8a). We observed a similar, albeit less dramatic, trend for LTR elements (Fig. 2g, *p* = 2.7 × e^−7^, Wilcoxon's rank-sum test). However, for SINE elements, there was no significant age difference observed between variable and common chromatin sites (Fig. 2h, *p* = 0.22, Wilcoxon's rank-sum test). DNA transposons that overlap variable chromatin show slight enrichment at older elements (Additional file 1: Figure S5b, *p* = 0.049, Wilcoxon's rank-sum test). However, DNA transposons contribute to a much smaller population of variable chromatin sites as compared to other classes of TEs (Fig. 2a–c, Additional file 1: Figure S5a). These results indicate that younger TEs, especially LINEs, display increased variation in regulatory potential across strains of mice and therefore may be involved in more recent adaptations of regulatory networks.

### Increased chromatin accessibility at younger LINEs

**Fig. 3:**
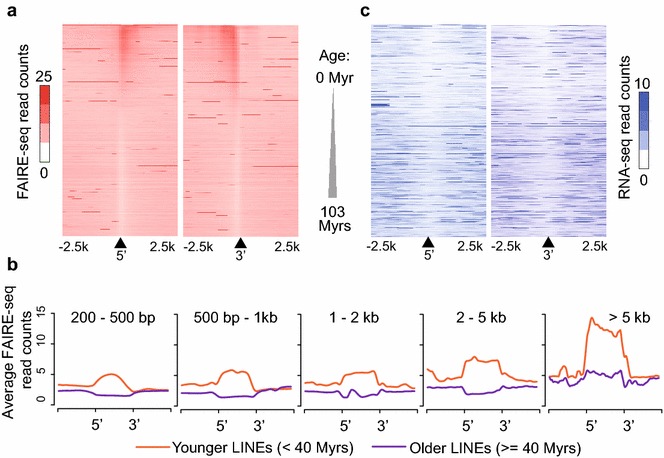
Differential chromatin accessibility profiles at younger and older LINEs. **a** Heatmap showing FAIRE-seq read counts from C57BL/6J mouse liver surrounding the 5′ and 3′ borders of LINEs sorted by their evolutionary age. *Black triangles* denote the 5′ or the 3′ end of LINEs, with counts extending ±2500 bp upstream and downstream. **b** Aggregate plots of average FAIRE-seq read counts upstream, downstream and within LINEs, stratified by size of LINEs. **c** Heatmap showing RNA-seq read counts from C57BL/6J mouse liver surrounding the 5′ and 3′ border of LINEs sorted by their evolutionary age. *Myrs* million years

In order to understand the regulatory roles of younger LINEs, we examined the chromatin accessibility differences at all LINEs ranked by their evolutionary age (Fig. 3a). To better examine the coverage of all mappable (but not necessarily unique) reads from FAIRE-seq data at repetitive elements, we mapped FAIRE-seq reads to the mouse genome using bowtie2 [39], which is capable of mapping non-unique reads from highly similar TE elements to a given subfamily of TE [40] (see “Methods” section). To examine chromatin accessibility differences among younger and older LINEs, we ranked all RepeatMasker-annotated LINEs by their evolutionary age and then plotted C57BL/6J liver FAIRE-seq read counts upstream and downstream of the annotated 5’ start and 3’ end of all LINEs (Fig. 3a). Interestingly, we found enriched chromatin accessibility at younger LINEs compared with older LINEs (Fig. 3a). To further examine the profiles of chromatin accessibility across entire LINE elements, we stratified LINEs into different groups based on their size and produced aggregate plots of the FAIRE-seq signal at LINEs and flanking regions (Fig. 3b). Consistent with the heatmap analysis (Fig. 3a), younger LINEs have higher chromatin accessibility compared with older LINEs, regardless of size (Fig. 3b). We also found that longer LINEs have more enriched chromatin accessibility compared with shorter ones (Fig. 3b), likely because the longer intact LINEs tend to be evolutionarily younger. Chromatin accessibly in another strain of mice, A/J, reveals a similar trend (Additional file 1: Figure S9). It has previously been shown that intact, longer, LINEs can be transcribed [20]. However, we did not detect increased RNA transcripts from younger LINEs compared with older LINEs (Fig. 3c). To ensure that the differential chromatin accessibility and uniform transcription profiles at younger vs older LINEs were not due to mapping biases, we repeated analysis of FAIRE-seq and RNA-seq enrichment at LINE families using TEtranscripts [41], a software package designed for including TEs in the analysis of sequencing datasets. This analysis supported our conclusions that differential chromatin accessibility exists at younger vs older LINEs but there is no differential transcription (Additional file 1: Supplementary methods, Figure S10). However, we cannot rule out the possibility that transcripts from TEs are subjected to posttranscriptional suppression that affects RNA stability [15]. Nevertheless, these results indicate that a group of evolutionarily younger LINEs have potential regulatory features in mouse liver, while not producing stable transcripts.

### Differential transcription factor binding sites at younger and older LINEs

**Fig. 4:**
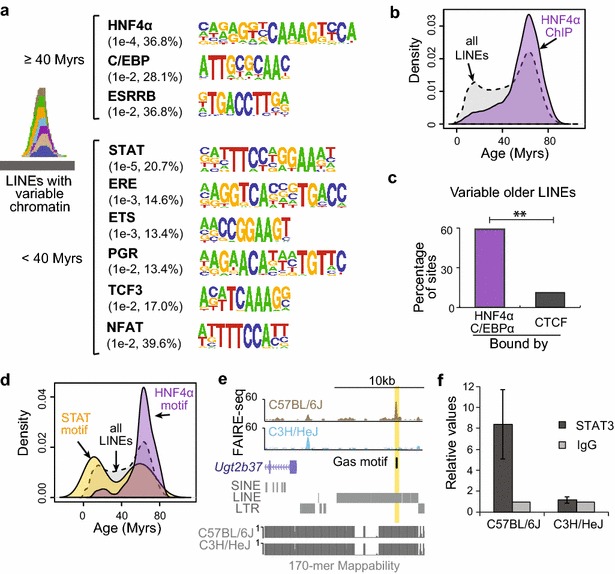
Specific TFs bind to younger and older LINEs. **a** Top known motifs found in variable chromatin sites overlapping older (*top*) or younger (*bottom*) LINEs. *Numbers* in parentheses represent *p*-values of enrichment of motif occurrence in the given set of sequences compared with background, and the percentage of sequences with the motif. **b** Age distribution of all LINEs as well as LINEs containing HNF4α ChIP-seq peaks. *Myrs* million years. **c** Percentage of older-LINE-associated variable sites bound by liver TFs (HNF4α and C/EBPα) or CTCF (**indicates *p* < 0.001, Fisher’s exact test). **d** Age distribution of all LINEs as well as LINEs containing STAT or HNF4α motif within accessible chromatin sites in C57BL/6J mouse liver. **e** Genome browser view of an L1Md_F2 located upstream of Ugt2b37. The GAS motif site is overlapping a site that is accessible in C57BL/6J but not C3H/HeJ. The 170-mer mappability tracks for the reference genome and the C3H/HeJ pseudo-genome are shown below. **f** Mean relative value of STAT3 ChIP-qPCR compared to IgG control of liver samples from C57BL/6J or C3H/HeJ mice. *Error bars* represent the SEM (*n* = 3)

TEs have been shown to contain transcription factor binding sites, and contribute to the evolution of the mammalian TF binding repertoire [24, 27, 42]. We examined the potential regulatory roles of LINEs by scanning for binding sites of known TFs in LINE-associated variable chromatin sites stratified by age (see “Methods” section). Intriguingly, we found that different TF binding motifs are enriched at sites overlapping older LINEs compared with those overlapping younger LINEs (Fig. 4a). The motif for HNF4α, a liver TF, is the top enriched motif in variable chromatin sites containing older LINEs (Fig. 4a). HNF4α ChIP-seq data from C57BL/6J liver [43] also validated the enrichment of HNF4α binding at older LINEs compared to younger ones (Fig. 4b; *p* < 2.2 × e^−16^, Wilcoxon's rank-sum test). In addition, the binding motif for another liver TF, C/EBPα, is also enriched at variable sites containing older LINEs (Fig. 4a). Notably, 59 % (67/114) of variable chromatin sites overlapping older LINEs are bound by the two liver TFs, HNF4α and/or C/EBPα. To serve as a control, we searched for the sites that are bound by CTCF [4], a non-liver-specific TFs. Compared with HNF4α and/or C/EBPα, we found only 11 % (12/114) of variable chromatin sites overlapping older LINEs to be bound by CTCF, indicating the important role of older LINEs in liver-specific transcription regulation (Fig. 4c, *p* = 7.5 × e^−15^, Fisher’s exact test).

Intriguingly, variable chromatin sites containing younger LINEs are most enriched for the binding motif of STAT proteins (Fig. 4a), which have been shown to play an important role in response to inflammation in liver [44]. In addition, we noticed that several other enriched motifs contain a half GAS motif (TTC or GAA), to which STATs can also bind [45, 46]. To further investigate the presence of specific TF binding at specific LINEs, we used the occurrence of motifs at accessible chromatin sites in C57BL/6J mouse liver as a predictor of binding [47]. Of the predicted HNF4α binding sites, 87 % (7209/8296) have HNF4α ChIP-seq peaks in mouse liver [43]. We furthermore found that compared with older LINEs that are enriched for HNF4α binding sites, younger LINEs at accessible chromatin regions are enriched for STAT binding sites (Fig. 4d; *p* = 2.3 × e^−9^, Wilcoxon's rank-sum test). To confirm the results, we performed chromatin immunoprecipitation (ChIP) using antibodies targeting STAT3, a member of the STAT protein family known to be active in the liver [48]. Using quantitative PCR (qPCR), we found that STAT3 binds to an L1Md_F2 element in a strain-specific manner (Fig. 4e, f). These results indicate that younger LINEs may play a role in the STAT-mediated immune response in the liver.

### TE-associated variable chromatin sites contribute to liver metabolic pathways

**Fig. 5:**
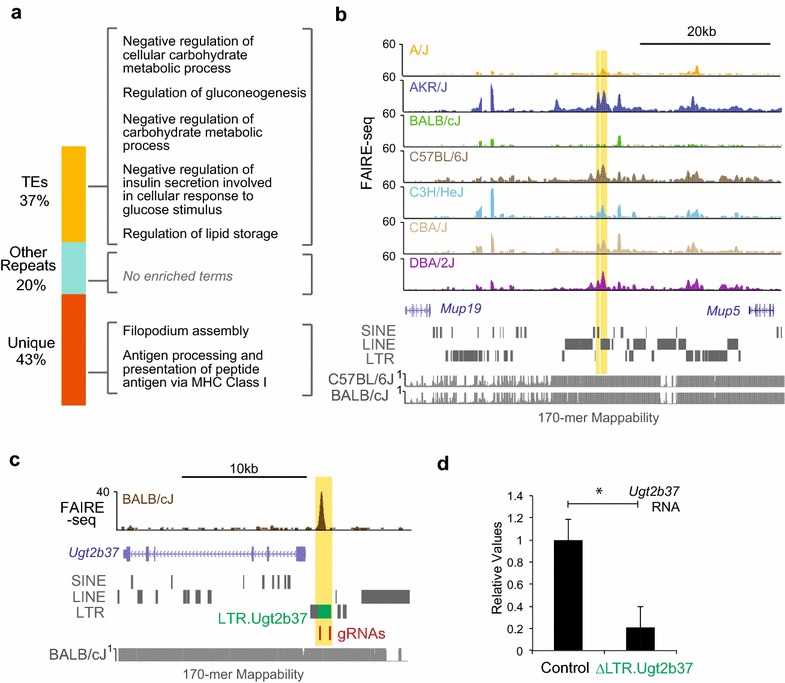
TE-associated chromatin variation impacts the expression of liver metabolic genes. **a** Top enriched biological processes of variable chromatin sites overlapping TEs, other repeats or non-repetitive (unique) sequences. The full list of enriched biological pathways is in Additional file 1: Table S3. Genomic coordinates of accessible chromatin sites were used as input for genomic regions enrichment of annotations tool (GREAT) analysis (see “Methods” section). **b** Genome browser view of TE-associated variable chromatin sites within the *MUP* locus. **c** Genome browser view of an LTR-associated variable chromatin site upstream of *Ugt2b37*. The guide RNAs designed for CRISPR-Cas9 deletion, and 170-mer mappability tracks of BALB/cJ pseudo-genome are shown below. **d** Quantitative polymerase chain reaction (qPCR) of *Ugt2b37* expression levels in control or ΔLTR.Ugt2b37 H2.35 cells (*indicates *p* < 0.05, Student’s *t* test)

To further investigate the impact of variable chromatin at TEs to phenotypic diversity among strains, we used the genomic regions enrichment of annotations tool (GREAT) [49] to identify enriched biological functions of accessible chromatin sites overlapping TEs. We found that variable chromatin sites with TEs are enriched in liver metabolic pathways, including gluconeogenesis, insulin secretion and lipid storage (Fig. 5a). Variable chromatin sites containing younger LINEs are enriched in the negative regulation of gluconeogenesis (Additional file 1: Table S3). In contrast, variable chromatin sites at unique sequences (without TE or other repeats) of the genome are only enriched for filopodium assembly and antigen processing pathways (Fig. 5a). In addition, variable chromatin sites overlapping other types of repeat show no enrichment for biological functions and comprise only a small percentage of variable chromatin sites. To serve as a control, we also searched for enriched biological processes in common chromatin sites with or without TEs. Not surprisingly, both groups of common chromatin sites are enriched for liver metabolic processes, including triglyceride metabolic process and cellular response to oxidative stress (Additional file 1: Table S3), indicating that these liver metabolic pathways are conserved and tightly regulated in all the strains. Taken together, these results suggest that the variation of chromatin accessibility among different strains is associated with liver metabolic pathways through specific TE sequences.

As an example, several TE-associated variable chromatin sites are found in the major urinary protein (MUP) gene locus. Two LINE-associated variable sites proximal to *Mup19* and *Mup5* are shown in Fig. 5b. MUP family proteins are expressed mainly in the liver and bind to small lipophilic molecules, including fatty acids [50]. MUPs have been shown to play important roles in glucose and lipid metabolism and are highly polymorphic in mice [50–52]. Our results here suggest that TEs are involved in this polymorphic feature of MUPs in the mouse genome.

To validate the function of TEs at variable chromatin sites in regulating nearby gene expression, we used the CRISPR-Cas9 system to generate deletion of TE sequences in H2.35 cells, a cell line derived from BALB/c hepatocytes. We first tested an LTR located 1 kb upstream of *UDP glucuronosyltransferase 2 family, polypeptide B37 (Ugt2b37)*, a member of UGT family (Fig. 5c). UGT gene family members encode enzymes in detoxification pathways and are upregulated in steatotic liver tissue from obese mice [53]. The deletion of this LTR leads to significant reduction in *Ugt2b37* expression (Fig. 5d, *p* = 0.03, Student’s *t* test). We further tested two additional variable LINEs and showed the deletion of each leads to the dysregulation of nearby metabolic genes. Deletion of an L1Md_F2 located 11 kb upstream of *Ugt2b37* also leads to significant reduction in *Ugt2b37* expression (Additional file 1: Figure S11a, *p* = 0.004, Student’s *t* test). We also deleted an Lx8 LINE element 6 kb downstream of *suppressor of defective silencing 3 homolog* (*Suds3)*. *Suds3* encodes a protein component of the SIN3 histone deacetylase (HDAC) corepressor complex, which has been shown to play a regulatory role in metabolic control in the liver [54]. Deletion of the Lx8 leads to increased expression of *Suds3*, which indicates a potential suppressor function of the Lx8 (Additional file 1: Figure S11b, *p* = 0.02, Student’s *t* test). These results validated that variable TEs contribute to the regulation of metabolic genes in liver cells.

**Fig. 6:**
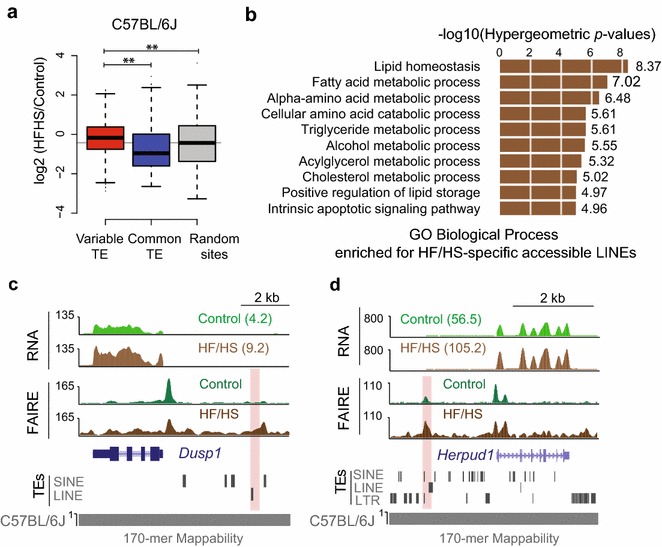
Variable TEs are associated with diet-induced gene expression changes. **a** Fold change (HF/HS over control) of C57BL/6J FAIRE-seq reads at variable TE sites, common TE sites or random accessible sites. *Box* *plots* show the median value, and* whiskers* show distribution of first and third quartile (**indicates *p* < 0.0001, Wilcoxon's rank-sum test). **b** Enriched biological processes of accessible LINEs specific for HF/HS-fed C57BL/6J mice (4339 regions). Genomic coordinates were used as input for GREAT analysis. **c**, **d** Genome browser views of LINE-associated diet-induced chromatin remodeled sites nearby diet-induced metabolic genes, *Dusp1* (**b**) and *Herpud1* (**c**). *Numbers* in parentheses for RNA-seq tracks represent FPKM values

Given that sites displaying chromatin variation at TEs are enriched in metabolic pathways, we hypothesized that variable TE sequences regulate nearby metabolic genes in response to diet. Our previous work has demonstrated that HF/HS diet leads to chromatin remodeling at regulatory regions in the liver [55]. We therefore examined chromatin accessibility differences in control-fed and HF/HS diet-fed C57BL/6J male mice from [55]. Intriguingly, TE-associated variable chromatin sites have increased accessibility in response to HF/HS diet, compared with common sites or random sites (Fig. 6a). Examining the accessibility of LINE elements that were unique to either diet condition revealed that accessible LINEs in HF/HS-fed mice, but not control-fed mice, are enriched for lipid metabolic pathways (Fig. 6b) and are proximal to metabolic genes with altered expression in response to HF/HS diet (Fig. 6c, d). These results indicate that TEs contribute to regulatory changes in the liver in response to diet.

### TE polymorphic variants contribute to regulatory variation across inbred strains

**Fig. 7:**
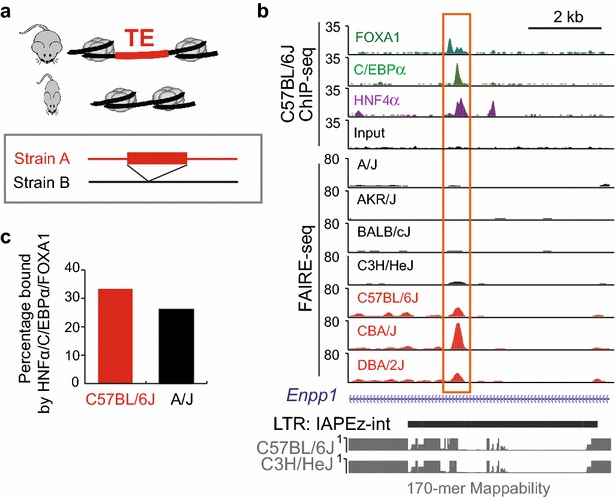
TE polymorphism accounts for a small percentage of chromatin variation. **a** A model of TE contribution to chromatin variation through TE polymorphism. **b** Genome browser view of a polymorphic LTR variant with variable chromatin accessibility. Strains with FAIRE-seq tracks* colored * in *red* contain the LTR element, while the strains in *black* do not have the LTR element. ChIP-seq tracks of three liver TFs and input from C57BL/6J mouse liver are also shown. **c** Percentage of polymorphic TE-associated variable chromatin sites shown to be bound by liver TFs in C57BL/6J or A/J mice livers

Given the widespread contribution of TEs to regulatory networks, we were further interested in characterizing the potential mechanisms responsible for TE-driven regulatory variation among different strains. One possible mechanism whereby TEs could contribute to chromatin accessibility variation is TE polymorphism—where a TE is present in one genome and not in another (Fig. 7a). A previous study has characterized TE polymorphism across 18 strains of mice, including the seven strains in our study [10]. Figure 7b shows an example of a polymorphic LTR variant associated with chromatin variation. The LTR element present in C57BL/6J, CBA/J and DBA/2J genomes [10] contains a strain-specific accessible chromatin region. Interestingly, the accessible chromatin site within the LTR also shows evidence of binding by liver TFs, including HNF4α, C/EBPα and FOXA1 (Fig. 7b). This region is within the intron of the *Enpp1* gene, which encodes a pyrophosphatase, and has been shown to be related to type 2 diabetes [56]. These results indicate that polymorphic TE-associated chromatin sites may play a strain-specific regulatory role for *Enpp1*. All together, we found approximately 30 % of polymorphic TE sites are bound by liver TFs (Fig. 7c), suggesting that these polymorphic TE-associated variable chromatin sites are playing regulatory roles. While we found that only 6 % (59/934) of the TEs that overlap with variable chromatin sites are polymorphic among the strains, this is likely an underestimate given the difficulty in genome assembly at repetitive regions of the genome [10].

### Differential DNA methylation at TEs contributes to regulatory variation across inbred strains

**Fig. 8:**
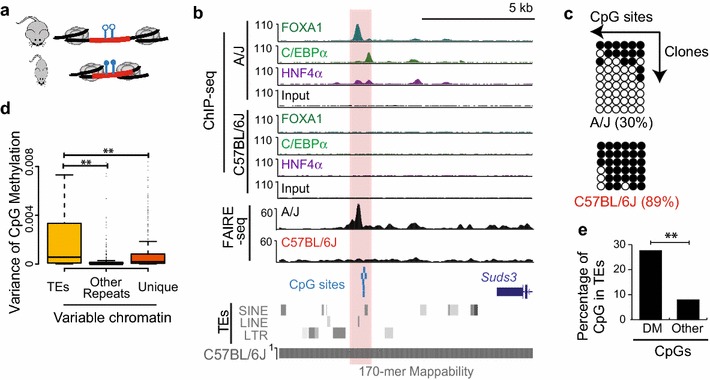
Differential DNA methylation at TEs can impact chromatin accessibility variation across inbred strains. **a** A model of TE contribution to chromatin variation through differential DNA methylation. **b** Genome browser view of differentially methylated TEs with variable chromatin accessibility. ChIP-seq tracks of three liver TF and input from C57BL/6J and A/J livers are also shown. CpG sites within the highlighted accessible chromatin sites are shown in *blue*. **c** Bisulfite sequencing in A/J and C57BL/6J liver of the highlighted region in **b**. *Filled circles* represent methylated CpGs, whereas* open circles* represent unmethylated CpGs. **d** *Boxplot* of CpG methylation variance among the seven strains within variable chromatin sites that contain TEs, other repeat elements or unique sequences. *Box* *plots* show the median value, and* whiskers* show distribution of first and third quartile. **e** Percentage of differential methylated (DM) vs other CpGs in TE sequences. Differential methylation was defined from RRBS data of 25 inbred strains (see “Methods” section) (**indicates *p* < 0.001, Fisher’s exact test)

It has been previously demonstrated that TEs are subject to regulation through epigenetic mechanisms, including DNA methylation and histone modifications [17]. In human somatic cells, DNA hypomethylation has been found within specific TE subfamilies that are associated with enhancer marks [19]. We therefore reasoned that TEs with differential chromatin accessibility not classified as polymorphic could be differentially regulated through epigenetic mechanisms, such as DNA methylation (Fig. 8a). An example of a TE-containing variable chromatin locus with negatively associated CpG methylation levels is shown in Fig. 8b. Interestingly, strain-specific (A/J vs C57BL/6J) binding of liver TFs [43] indicates that this region is differentially bound by liver TFs as well (Fig. 8b). Bisulfite sequencing at the region highlighted in Fig. 8b in livers from A/J and C57BL/6J revealed differential methylation of this region (Fig. 8c). To examine the impact of differential methylation at TEs to chromatin variation across the genome, we utilized reduced representation bisulfite sequencing (RRBS) data from liver tissue of the same strains of mice [57]. Interestingly, variable chromatin sites at TEs have a greater degree of DNA methylation variation across strains as compared to variable chromatin sites at other regions (Fig. 8d). These results indicate that differential epigenetic suppression of TEs contributes to chromatin accessibility variation across the strains.

To further validate that the epigenetic variation at TEs in liver is not only restricted to the seven inbred strains of mice, we compared the CpG methylation levels from livers of 25 inbred mouse strains [57]. Consistent with the results presented above, the differentially methylated (DM) CpG sites among inbred strains are significantly enriched for TEs compared to other CpG sites (Fig. 8e, *p* < 2.2 × e^−16^, Fisher’s exact test). These results suggest that widespread chromatin variation at TEs is a general feature in mouse liver.

## Discussion

While previous studies have identified a genetic component to chromatin variation [7], the mechanisms underlying the majority of chromatin variation have remained unexplained. We report here that TEs are a major contributor to chromatin variation in liver tissue and furthermore that TE-driven chromatin variation is important for metabolic phenotypes.

We have previously shown that variation in chromatin accessibility across three strains of mice in response to diet depends on genetic factors [55, 58]. We have now extended our study to a total of seven inbred strains that have significant variability in liver phenotypes in response to a HF/HS diet. The variability of the phenotype in these mice resembles the diversity of diet response in humans [59]. Although accessible chromatin sites are less likely to overlap TEs in liver tissue in general (Additional file 1: Figure S4a, b), we found that chromatin sites with higher variability in different strains are enriched for TEs, specifically evolutionarily younger LINEs. We furthermore demonstrated that strains with higher accessibility for a given young L1Md subfamily also display higher accessibility for other young L1Md subfamilies. One explanation for this is that certain strains have less faithful silencing of younger LINEs compared with others. Further studies examining the strain-specific regulation of young LINEs will be enlightening. In addition to potential long-range effects, variability of chromatin accessibility at TEs might also be influenced by local genetic variation, as indicated by the regions where genotype and chromatin accessibility correspond (Additional file 1: Supplementary methods, Figure S7b, Figure S14).

Epigenetic variability can occur both inter-strain and inter-individual. In our study, we used duplicates of each strain of mice for chromatin accessibility profiling and employed a computational pipeline (see “Methods” section) to identify reproducible chromatin variation among different strains of mice. A previous study on C57BL/6J mice showed inter-individual variation of DNA methylation at TEs [60]. We used the 356 regions identified as inter-individual differentially methylated regions [60] and found less than 1 % (16/2539) of our variable sites contain inter-individual variability, indicating that the majority of the variable chromatin sites we identified represent sites of variability among different strains of mice.

TEs have been shown to play an important role in expanding the TF binding repertoire during mammalian evolution [24, 27]. Supportive of this, we found that younger and older LINEs have differential chromatin accessibility and are bound by different TFs. Evolutionarily older LINEs are enriched for binding sites of liver regulatory factors (HNF4α and C/EBPα), indicating their important regulatory roles in the liver. In contrast, younger LINEs are enriched for the binding sites of TFs involved in immune response, such as STATs. The relationship between STAT and TEs is intriguing; a recent study demonstrated that specific TEs play a functional role in immune pathways in human HeLa cells [21]. It is possible that the variable sites uncovered by our studies also contribute to immune pathways regulated by STAT proteins. STATs have been show to be involved in the development of hepatosteatosis [61], which can also be induced by HF diet [62]. Given that these STAT-bound LINEs are at variable chromatin sites, STAT binding to the young LINEs could be a source of chromatin variation. Importantly, our results indicate that specific LINE elements of different evolutionary age have contributed unique elements to regulatory networks.

We further investigated possible mechanisms of chromatin variation at TEs. TE polymorphism explains at least 6 % of the TE contribution to chromatin variation. Previous work has shown that less than 10 % of these structural variants result in detectable gene expression changes [10]. However, we found approximately 30 % of polymorphic TE sites to be bound by liver TFs, indicating that they play a regulatory role in liver. This discrepancy may due to the high stringent threshold used in the previous work [10]. It is also possible that liver-TF-bound sites are not directly regulating nearby gene targets [63].

Epigenetic mechanisms, such as DNA methylation, have been shown to play an important role in suppression of TE activity in somatic cells [17]. We show here that variation of CpG methylation at TEs contributes to chromatin accessibility variation. DNA hypomethylation at specific TEs has been shown to be associated with enhancer activity [19]. Therefore, these TE-associated chromatin sites may have differential enhancer activity in different strains. Future studies on histone modifications may explain more of the impact of TE epigenetic regulation on chromatin accessibility.

Our finding that TEs contribute to chromatin variation and metabolic gene regulation suggests that the phenotypic diversity observed across the strains is at least partially due to the regulatory role of TEs. One of the classical models of TE contribution to phenotypic diversity is the *agouti viable yellow* (*A*^*vy*^) gene, for which a TE exists upstream of the *A*^*vy*^ gene [64]. Variation of DNA methylation at this TE regulates the expression of the *A*^*vy*^ gene and therefore leads to differential coat color and obesity susceptibility [64, 65]. Further experimental validation on the TE-associated variable chromatin sites may lead to the identification of more examples like this. Studies in different tissue types and disease systems may further reveal the impact of TEs to phenotypic diversity.

## Conclusions

In summary, our study has revealed that specific classes of TEs, especially younger LINEs, can impact chromatin accessibility variation in liver of different inbred strains. We further demonstrate that TEs regulate tissue-specific genes which may result in downstream phenotypic diversity.

## Methods

### Animal

Mice were obtained from The Jackson Laboratory and were bred at the University of California, Los Angeles. Male A/J, AKR/J, BALB/cJ, C57BL/6J, C3H/HeJ, CBA/J and DBA/2J mice were maintained on a chow diet (Ralston Purina Company) until 8 weeks of age. Then they were given a high-fat, high-sucrose diet (Research diets D12266B, 16.8 % kcal protein, 51.4 % kcal carbohydrate and 31.8 % kcal fat) for 8 weeks. During the feeding period, body fat percentage was tracked as described previously [28]. Mice were then humanely euthanized and livers were harvested. All animal study protocols in this study were approved by the Institutional Care and Use Committee (IACUC) at University of California, Los Angeles and by the Institutional Care and Use Committee (IACUC) at the City of Hope.

### Phenotypic characterization of mice

Hematoxylin and eosin (H&E) staining and Oil red O staining were performed on liver sections by the Pathology Core at the City of Hope using standard procedures.

### FAIRE-seq and alignment

Formaldehyde-assisted isolation of regulatory elements (FAIRE) was performed on flash frozen liver tissues from two biological replicates in each strain as previously described [29]. Isolated FAIRE DNA fragment from each sample was barcoded and sequenced on the Illumina HiSeq 2500 to produce 100 × 100 bp paired-end reads.

In order to eliminate the mapping biases caused by inter-strain sequence variation, we first generated a pseudo-genome for each non-reference strains by introducing SNPs from each strain into the reference mouse genome (mm9) [33]. We then mapped FAIRE-seq reads from each replicate to the appropriate pseudo-genome using bowtie1 [66] and only reads that could be mapped to single location in the genome were retained. Aligned reads were further filtered to exclude improperly paired reads and PCR duplicates. Overall, we obtained around 17 million uniquely mapped non-duplicate reads in each sample (Additional file 1: Table S1). Wiggle tracks were generated for visualization on the UCSC Genome Browser [67].

For the analysis of FAIRE-seq and RNA-seq coverage at LINEs (Fig. 3), we mapped reads to the reference genome using bowtie2 with the local alignment option [39], as described previously [40]. Unlike bowtie1 with unique mapping mode, the bowtie2 alignment method keeps reads with multiple alignments and reports the best alignments [39]. Therefore, the reads from highly similar TE elements can be mapped to a given subfamily of TE.

### Mappability score

In order to mitigate mapping biases, we generated mappability scores for the reference (C57BL/6J) and non-reference pseudo-genomes. We used the genome multitool (GEM) mapper [68] to generate mappability scores. The average length of paired-end FAIRE-seq fragments for the seven strains was 170 ± 3 bp (mean ± standard deviation). Therefore, we generated 170-mer mappability scores with up to two mismatches allowed. The mappability score (*M*) measures how often the sequence found at the particular location will align within the whole genome. *M* = 1 means unique match in the genome, *S* = 0.5 means two matches in the genome, and so on. All the tracks shown here are in the form of signals ranging from 0 to 1.

### Accessible chromatin detection and analysis

To identify accessible chromatin sites from FAIRE-seq reads for each library, F-seq was used with default parameters and a 400 bp feature length [34]. To find reproducible peaks across replicates, we utilized the irreproducible discovery rate (IDR) framework [35]. To obtain a union set of accessible chromatin sites from the seven strains, we used the mergeBed function with default parameters [69].

To identify variable chromatin sites among different strains, we first counted the FAIRE-seq reads from each FAIRE-seq library at the union set of accessible chromatin sites. We normalized the read counts using quantile normalization [70]. We then used DESeq [36] to identify variable chromatin sites among the seven strains, as has been applied previously [7]. We ranked the accessible chromatin sites by adjusted *p*-values from DESeq. The 5 % with smallest adjusted *p*-values were considered as variable chromatin sites, whereas the 5 % with biggest adjusted *p*-values were considered as least variable (common) chromatin site among the seven strains (Additional file 1: Figure S2).

### Association between SNP genotype and chromatin accessibility

The correlation of FAIRE-seq signal and local sequence variation (Additional file 1: Figure S3) was analyzed as previously described [7]. Briefly, we translated the genotypes for all the seven strains at a certain SNP into a vector and evaluated the correlation of this vector to FAIRE-seq read counts at the overlapping accessible chromatin site by linear regression.

### Identification of TE-associated chromatin sites

To identify accessible chromatin sites at TE sequences, we used intersectBed [69] to find the accessible chromatin sites that overlap with TEs as annotated by RepeatMasker [37] for the mouse genome (mm9). The age of TEs was calculated as: age = divergence/substitution rate, as previously described [27]. The divergence rates (number of mismatches) for all TEs were obtained from the RepeatMasker annotation file [37]. We used the substitution rates as 4.5 × 10^−9^ per site per year for the mouse genome [11, 27].

### Motif scanning

To characterize TF motifs in LINEs, we used HOMER (version 4.8) (findMotifsGenome.pl) [71] to identify motifs of known TFs in variable chromatin sites containing younger (<40 million years (Myrs) or older LINEs (≥40 Myrs) as compared to random sequences with matched GC %. Motifs with *p*-value of enrichment less than 0.01 that occurred in more than 10 % of the target sequences were selected. Highly similar motifs were combined by using joinmotifs tool [72], and only one of the similar motifs is reported. HOMER was further used to scan for occurrences (scanMotifGenomeWide.pl) [71] of the HNF4α and STAT motif genome wide. Putative binding sites were defined by motif occurrences within accessible chromatin regions identified in C57BL/6J mouse liver, similar to what has been reported before [47].

### ChIP-Quantitative PCR

Chromatin immunoprecipitation (ChIP) was performed with an anti-STAT3 antibody (sc-482X, Santa Cruz Biotechnology) and IgG control using standard ChIP protocols. Fragmented chromatin was assessed for enrichment at specific sites by quantitative PCR quantitation. The ΔΔCt method was utilized to evaluate enrichment of target DNA and normalized to input DNA. qPCR primer sequences at the L1Md_F2 are in Additional file 1: Table S4. Based on in silico PCR, the primer set can bind to seven L1s in the genome. However, six of the potentially targeted regions contain STAT motif and display similar chromatin accessibility variation as shown in Fig. 4e.

### CRISPR-Cas9 genomic deletion

For each TE tested, two guide RNAs (gRNAs) were designed to generate specific deletion of the TE sequence in H2.35 cells, a cell line derived from BALB/c hepatocytes. All gRNAs (Additional file 1: Figure S12, Table S4) were verified to be unique targets in the mouse genome by using BLAT against mouse reference genome. We also avoided any gRNA targets that contained annotated SNPs in BALB/cJ mice. gRNA oligos were cloned into pSpCas9(BB)-2A-GFP and pSpCas9(BB)-2A-Puro vectors following a published protocol [73]. pSpCas9(BB)-2A-GFP (PX458) and pSpCas9(BB)-2A-Puro (PX459) V2.0 were gifts from Feng Zhang (Addgene plasmid #48138, #62988). H2.35 cells were co-transfected (Invitrogen, Lipofectamine 2000) with both gRNA constructs and placed under puromycin (1 μg/ml) selection for 3 days. Control cells were co-transfected with vectors without gRNA insertions. From these cells and control cells transfected with pSpCas9(BB)-2A-GFP and pSpCas9(BB)-2A-Puro, genomic DNA (Epicentre, Quickextract) and RNA were extracted (Trizol, Life Technologies) as recommended by the manufacturer. Genomic deletion was verified by PCR using flanking primer pairs at the expected deletion site (Additional file 1: Figure S12, Table S4). Reverse transcription quantitative PCR (RT-qPCR) was performed to determine the expression change in nearby gene(s) (primer sequences are in Additional file 1: Table S4).

### Bisulfite Sanger sequencing

Genomic DNA from liver tissue was bisulfite-treated according to the manufacturer’s instructions (EpiTect Bisulfite Kit, QIAGEN, USA). Converted genomic DNA was used for PCR (primer sequences are in Additional file 1: Table S4). Purified PCR products were cloned into pDrive Cloning vector (PCR Cloning Kit, QIAGEN, USA). White colonies were selected through blue/white screening and analyzed with Sanger sequencing.

### DNA methylation data

DNA methylation data from reduced representation bisulfite sequencing were obtained from GEO (accession number GSE67507 [57]). Similar to previous analyses [57], only CpG sites were included for analysis. For simplicity, we deleted the small amount of polymorphic CpGs in the seven strains from the methylation data. Differentially methylated (DM) regions were identified as those with a variance greater than 0.05 and range of methylation differences greater than 0.75.

### Gene ontology (GO) analysis

In order to investigate the enriched biological function of the genes nearby accessible chromatin sites, we used genomic coordinates (UCSC mm9) of accessible chromatin sites as input for genomic regions enrichment of annotations tool (GREAT) version 3.0.0 [49]. Gene regulatory regions were defined using default parameters (5 kb upstream, 1 kb downstream and up to 1000 kb distal) and included significant associations for “GO Terms Biological Process”. Only terms that were below a false discovery rate (FDR) of 0.01 were reported.

### RNA-seq and ChIP-seq data

RNA-seq data from livers of C57BL/6J and A/J mice fed with HF/HS diet were obtained from GEO (accession numbers GSE55581 [55] and GSE75984 [58]). ChIP-seq sites of liver TFs (HNF4α, C/EBPα, and FOXA1) from C57BL/6J and A/J liver tissues were downloaded from ArrayExpress (accession number E-MTAB-1414 [43]). CTCF ChIP-seq sites were obtained from GEO (accession number GSM918715 [4]).

## Additional files

10.1186/s13072-016-0078-0 **Figure S1.** Phenotypic diversity in different inbred strains. **Figure S2.** Chromatin variability across inbred strains of mice. **Figure S3.** Association between chromatin variation and SNPs. **Figure S4.** Accessible chromatin sites and TE sequences. **Figure S5.** DNA transposons and chromatin accessibility variation. **Figure S6.** Differential accessibility at young L1Md subfamilies across different strains. **Figure S7.** Chromatin accessibility at younger L1Md subfamilies in recombinant inbred strains. **Figure S8.** Chromatin variability and age of LINE subfamilies. **Figure S9.** Differential chromatin accessibility profile at younger and older LINEs in A/J mice liver. **Figure S10**. Accessibility and transcription of LINE subfamilies. **Figure S11**. CRISPR-Cas9 deletion of additional TEs. **Figure S12.** Guide RNA and genotyping primers used for CRISPR-Cas9 genome editing. **Figure S13.** Genotyping for TE deletions. **Figure S14.** Example of an eQTL that associated with variable chromatin accessibility at a LTR. **Table S1.** Summary of FAIRE-seq data sets in all the strains in this study. **Table S3.** Enriched biological process from GREAT analysis of accessible chromatin sites. **Table S4.** Sequences used in this study.
10.1186/s13072-016-0078-0 Counts of TEs in subfamilies.

## Abbreviations

TE: transposable element
FAIRE: formaldehyde-assisted isolation of regulatory elements
HF/HS: high fat, high sucrose
SNP: single nucleotide polymorphism
LINE: long interspersed nuclear element
SINE: short interspersed nuclear element
LTR: long terminal repeat
TF: transcription factor
TG: triglyceride
H&E: hematoxylin and eosin
Adi1: acireductone dioxygenase 1
Lipc: lipase, hepatic
Myrs: million years
HNF4α: hepatocyte nuclear factor 4 alpha
C/EBPα: CCAAT/enhancer binding protein alpha
FOXA1: forkhead box A1
STAT: signal transducers and activators of transcription
GAS: gamma-activated sequence
Ugt2b37: UDP glucuronosyltransferase 2 family, polypeptide B37
Enpp1: ectonucleotide pyrophosphatase/phosphodiesterase 1
Suds3: suppressor of defective silencing 3 homolog (*S. cerevisiae*)
Dusp1: dual-specificity phosphatase 1
Herpud1: homocysteine-inducible, endoplasmic reticulum stress-inducible, ubiquitin-like domain member 1
FPKM: fragments per kilobase million
DM: differentially methylated
