# Supplementary material for: Chromatin variation associated with liver metabolism is mediated by transposable elements
Source: Epigenetics Chromatin. 2016 Jul 8;9:28. doi: 10.1186/s13072-016-0078-0 (PMC4939004; doi:10.1186/s13072-016-0078-0)
Supplement: Supplementary file 1 — 10.1186/s13072-016-0078-0 Figure S1. Phenotypic diversity in different inbred strains. Figure S2. Chromatin variability across inbred strains of mice. Figure S3. Association between chromatin variation and SNPs. Figure S4. Accessible chromatin sites and TE sequences. Figure S5. DNA transposons and chromatin accessibility variation. Figure S6. Differential accessibility at young L1Md subfamilies across different strains. Figure S7. Chromatin accessibility at younger L1Md subfamilies in recombinant inbred strains. Figure S8. Chromatin variability and age of LINE subfamilies. Figure S9. Differential chromatin accessibility profile at younger and older LINEs in A/J mice liver. Figure S10. Accessibility and transcription of LINE subfamilies. Figure S11. CRISPR-Cas9 deletion of additional TEs. Figure S12. Guide RNA and genotyping primers used for CRISPR-Cas9 genome editing. Figure S13. Genotyping for TE deletions. Figure S14. Example of an eQTL that associated with variable chromatin accessibility at a LTR. Table S1. Summary of FAIRE-seq data sets in all the strains in this study. Table S3. Enriched biological process from GREAT analysis of accessible chromatin sites. Table S4. Sequences used in this study. [file 13072_2016_78_MOESM1_ESM.pdf]

Supplemental Materials for

Chromatin variation driven by transposable elements contributes to liver metabolism

Juan Du, Amy Leung, Candi Trac, Michael Lee, Brian W. Parks, Aldons J. Lusis, Rama Natarajan, Dustin E. Schones
Correspondence to: dschones@coh.org (D.E.S)

Contents:

Supplementary Methods
Figures S1 to S14
Tables S1 to S4 (Table S2 in a separate file)

Supplementary Methods:

Alignment of TE families from sequencing reads

In order to assess TEs as families, including reads that cannot be mapped uniquely to a single location in the genome, we utilized TE transcripts [1], a package developed for assessing both transposable elements and other transcripts.

FAIRE-seq and alignment for recombinant inbred strains

Two recombinant inbred strains, BXH2/TyJ and BXH19/TyJ, derived from C57BL/6J and C3H/HeJ, were fed and maintained as the other inbred strains (Methods). FAIRE-seq was performed as described (Methods). FAIRE-seq reads were aligned to the reference genome (mm9) and C3H/HeJ pseudo-genome. Similar results were obtained using either alignment strategy.

Identification of single nucleotide variants

Single nucleotide variants (SNVs) for BXH2/TyJ, BXH19/TyJ and C3H/HeJ are identified using 100x100bp paired-end FAIRE-seq reads. Sequencing reads were first processed by Btrim to remove adapters and low quality regions [2]. The trimmed reads were then aligned to reference genome (mm9) by using NovoAlign (http://novocraft.com), an aligner with high accuracy. Then we used The Genome Analysis Toolkit (GATK) [3] to identify SNVs in each strain. After identifying the SNVs, we compiled the SNVs from the four strains and converted the genotype information into a vector. For example, if C57BL/6J and BXH2/TyJ share the same genotype (while BXH19/TyJ and C3H/HeJ share a different genotype), we assign the genotype vector at the SNV locus as 0011 (where strains are in the order of BXH2/TyJ, BXH19/TyJ and C3H/HeJ). Then we made haplotype blocks by merging the SNVs that neighbor each other and share the same genotype vector.

References for Supplementary Methods:

1. Jin Y, Tam OH, Paniagua E, Hammell M: **TEtranscripts: a package for including transposable elements in differential expression analysis of RNA-seq datasets.** *Bioinformatics* 2015, **31:**3593-3599.

2. Kong Y: **Btrim: A fast, lightweight adapter and quality trimming program for next-generation sequencing technologies.** *Genomics* 2011, **98:**152-153.

3. McKenna A, Hanna M, Banks E, Sivachenko A, Cibulskis K, Kernytsky A, Garimella K, Altshuler D, Gabriel S, Daly M, DePristo MA: **The Genome Analysis Toolkit: a MapReduce framework for analyzing next-generation DNA sequencing data.** *Genome Res* 2010, **20:**1297-1303.

4. Marco-Sola S, Sammeth M, Guigo R, Ribeca P: **The GEM mapper: fast, accurate and versatile alignment by filtration.** *Nat Methods* 2012, **9:**1185-1188.

Figure S1

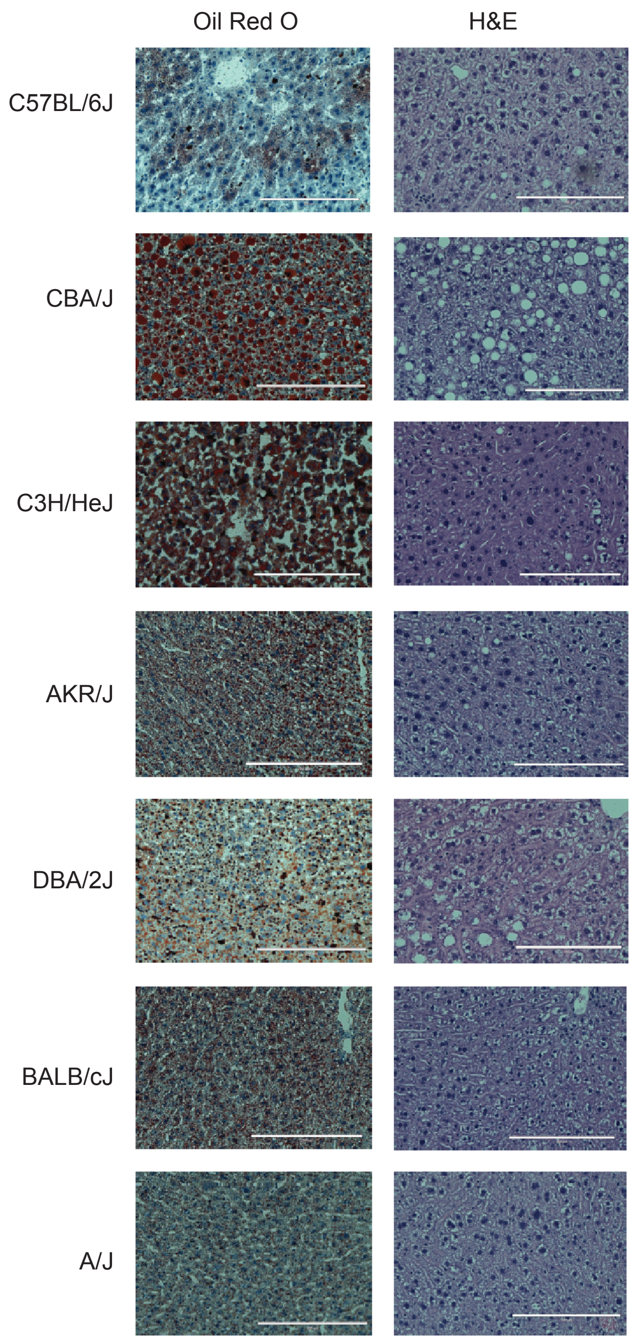

Figure S1. Phenotypic diversity in inbred strains. Oil Red O and H&E staining of liver sections from mice after eight weeks of HF/HS diet. Images were taken by Olympus IX51 inverted microscope (Magnification: 40X).

Figure S2

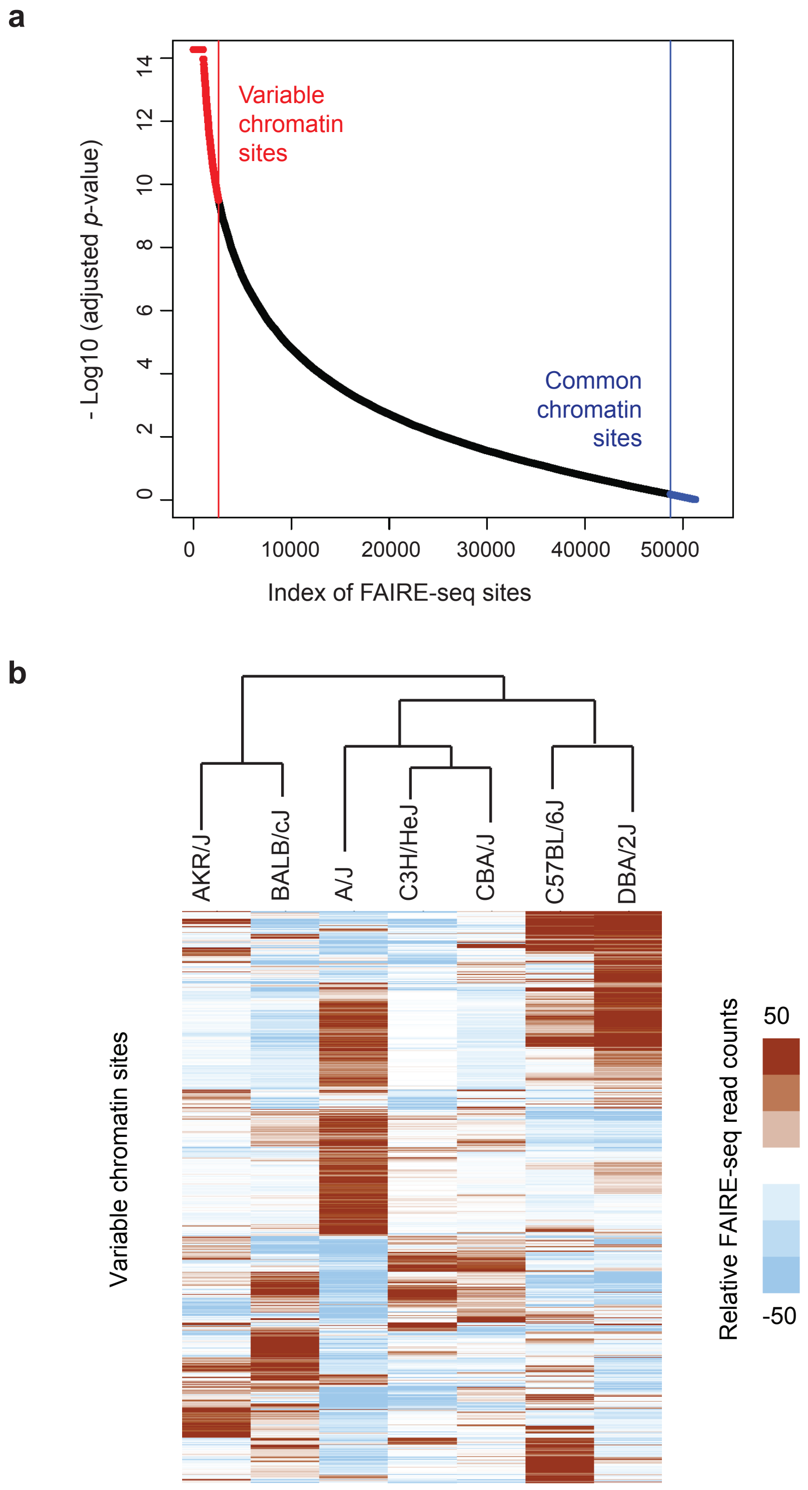

Figure S2. Chromatin variability across strains of mice. (**a**) Distribution of *p*-values of each FAIRE peak from DEseq analysis. (**b**) Unsupervised hierarchical clustering of relative FAIRE-seq read counts at variable chromatin sites (red data points in **a**) across seven strains of mice. The read counts from different strains at each chromatin sites were center on the median.

Figure S3

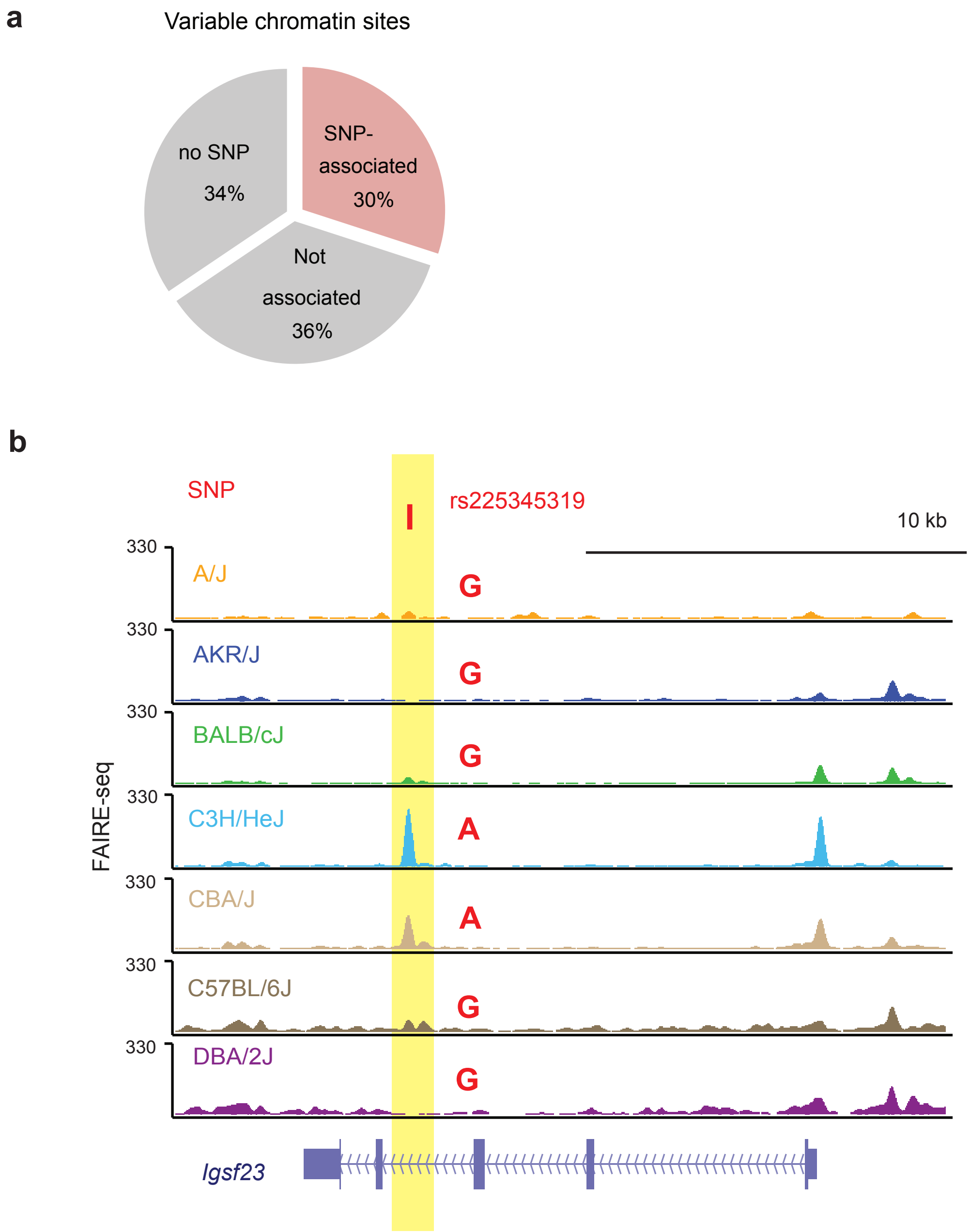

Figure S3. Association between chromatin variation and SNPs. (**a**) Fraction of most variable chromatin sites associated with underlying SNPs. (**b**) Genome browser view of a variable chromatin site associated with SNP rs225345319 in the 7 strains.

Figure S4

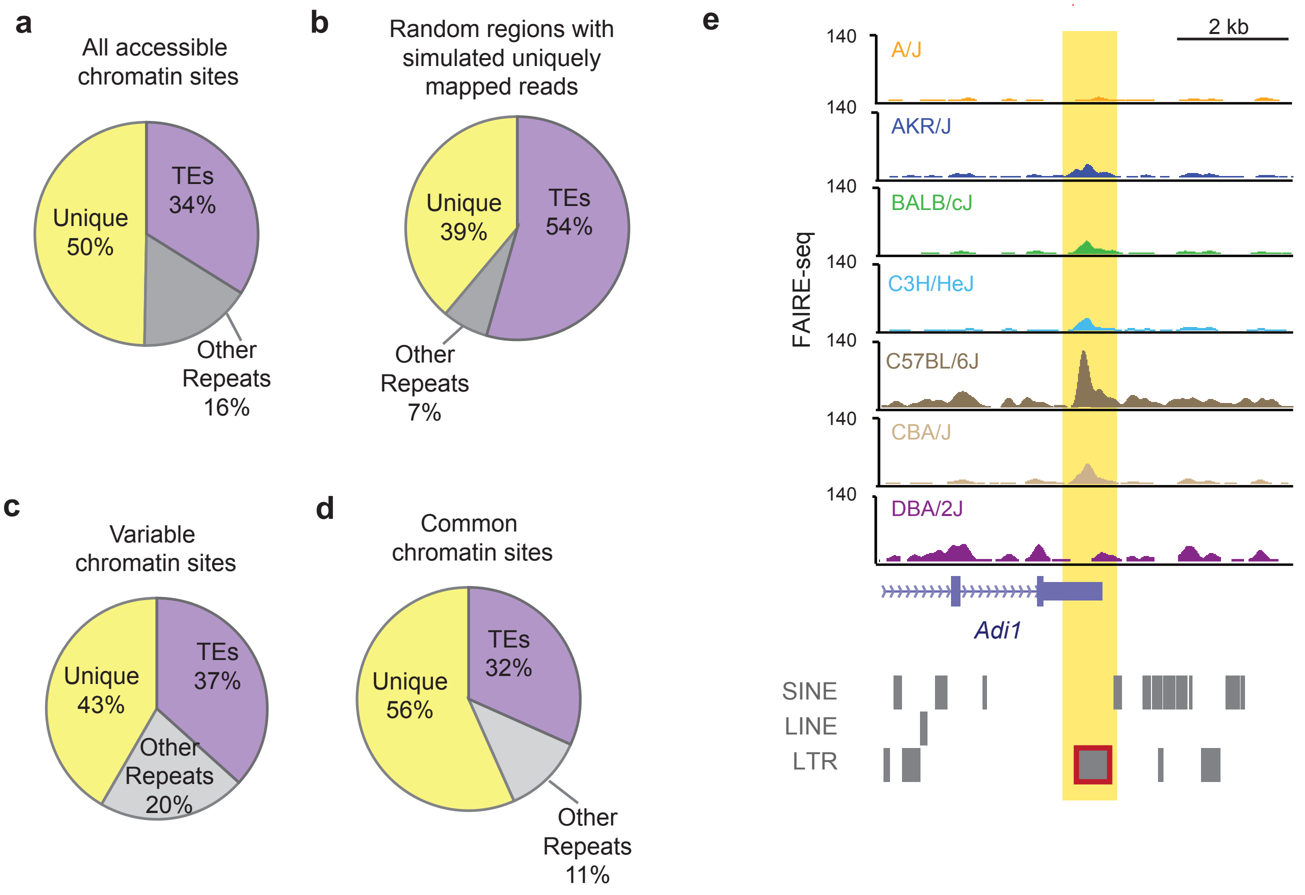

Figure S4. Accessible chromatin sites and TE sequences. (**a, b**) Proportion of (**a**) all accessible chromatin sites and (**b**) random genomic regions that overlap with TEs, other repeat elements (RepeatMasker annotated, non-TE elements), or non-repetitive (unique) sequences. (**c, d**) Proportion of (**c**) variable and (**d**) common chromatin sites that overlap with TEs, other repeat elements, or unique sequences. (**e**) Genome browser view of variable chromatin site in Fig. 1**b** coincides with a LTR. Shown are the seven FAIRE-seq wiggle tracks, RefSeq genes, RepeatMasker TEs.

Figure S5

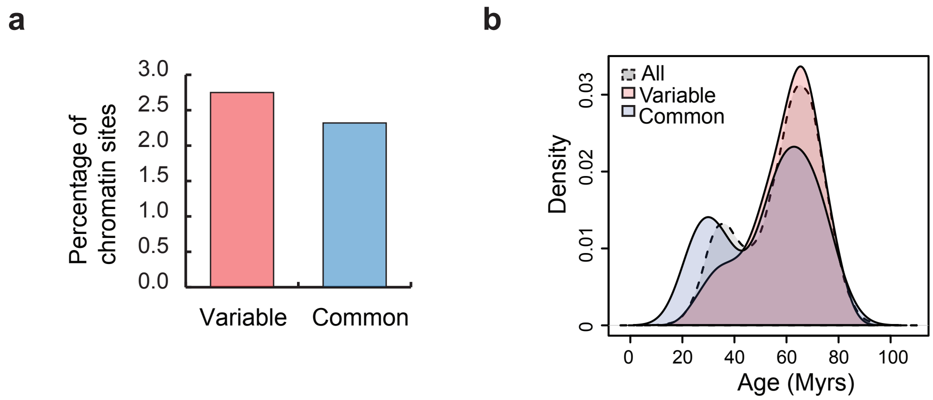

Figure S5. DNA transposons and chromatin accessibility variation. (**a**) Percentage of variable and common chromatin sites overlapping DNA transposons. (**b**) Age distribution of all DNA transposon, DNA transposon at variable or common chromatin sites. Myrs: million years.

Figure S6

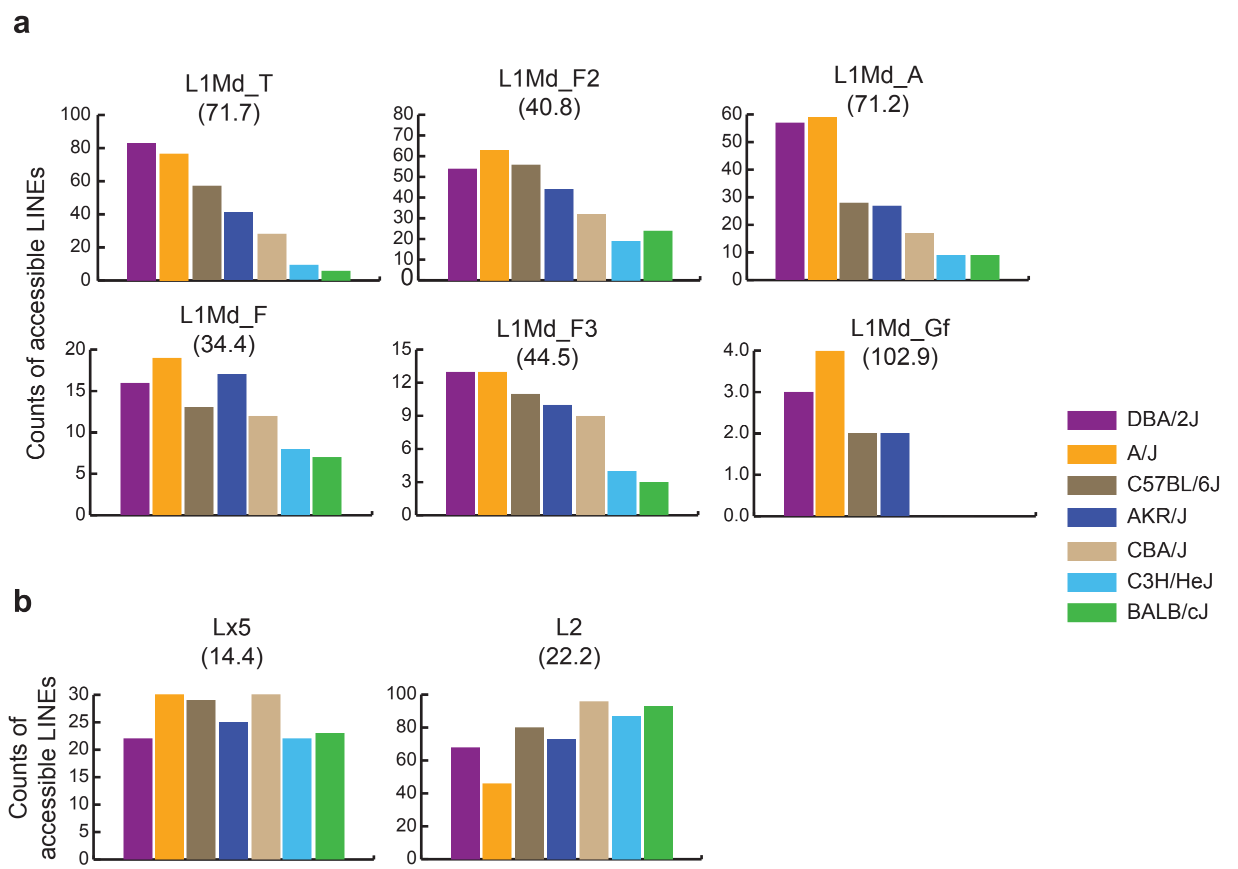

Figure S6. Differential accessibility at young L1Md subfamilies across different strains. (**a, b**) Counts of accessible LINEs in (**a**) younger LINE and (**b**) older subfamilies (the age of Lx5 and L2 are 48 and 66 million years, respectively) in liver of seven strains. Chromatin accessibility at older LINEs is more uniform than that of younger L1Mds. Coefficient of variation for the counts of each subfamily is shown in parentheses below the name.

Figure S7

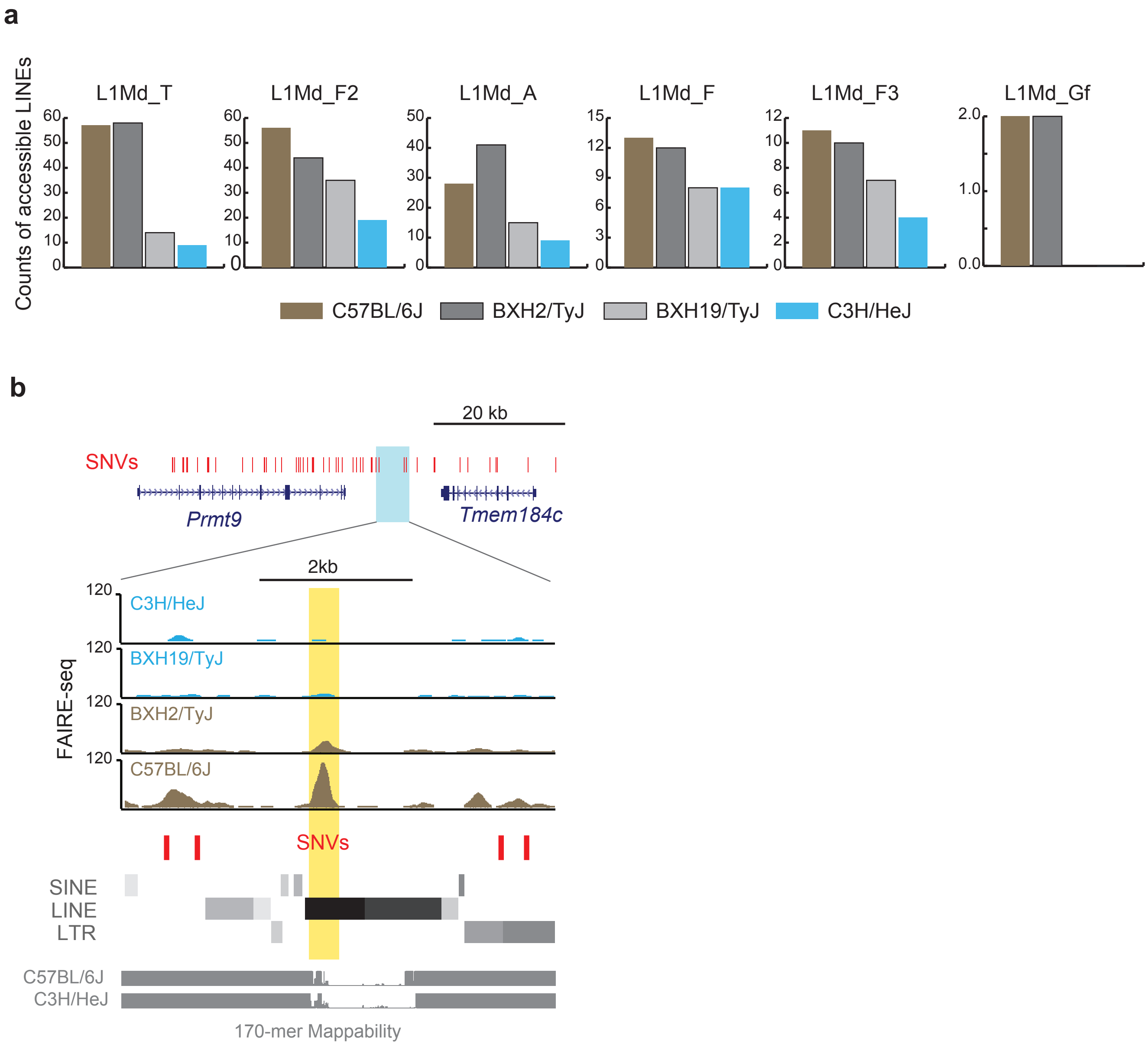

Figure S7: Chromatin accessibility at younger L1Md subfamilies in recombinant inbred strains. (**a**) Counts of accessible LINEs in liver of two recombinant inbred strains, and the parental strains. BXH2/TyJ have similar accessibility at young LINEs compared to C57BL/6J, while BXH19/TyJ is more similar to C3H/HeJ. (**b**) Example of a LINE that is accessible in C57BL/6J and BXH2/TyJ (but not C3H/HeJ and BXH19/TyJ) within regions where C57BL/6J and BXH2/TyJ share a genotype at the locus, while C3H/HeJ and BXH19/TyJ share a different genotype. Shown are the FAIRE-seq wiggle tracks, RefSeq genes, RepeatMasker TEs, SNVs within the haplotype block and 170-mer mappability tracks.

Figure S8

a

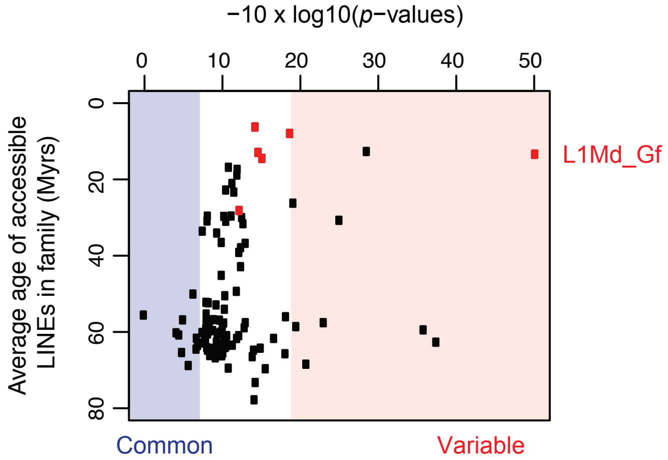

b

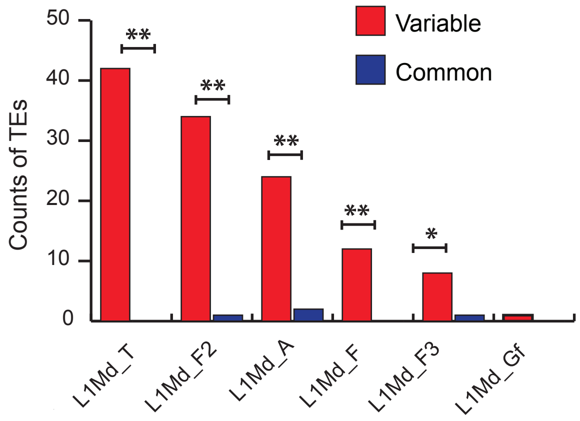

Figure S8. Chromatin variability and age of LINE subfamilies. (**a**) Variability of LINE families ranked by the average age of LINEs in the family. Variability was measured by the adjusted p-values from DEseq analysis (Figure S2a). L1Md subfamilies of LINEs are colored in red. (**b**) Fig. 2d with L1Md_Gf. Numbers of variable (red) or common (blue) chromatin sites overlapping L1Md subfamilies (*$p < 0.05$, **$p < 0.001$, Fisher's exact test). Although L1Md_Gf subfamily showed highest variability, only a small number of L1Md_Gf are at variable chromatin sites compared to other L1Md families.

Figure S9

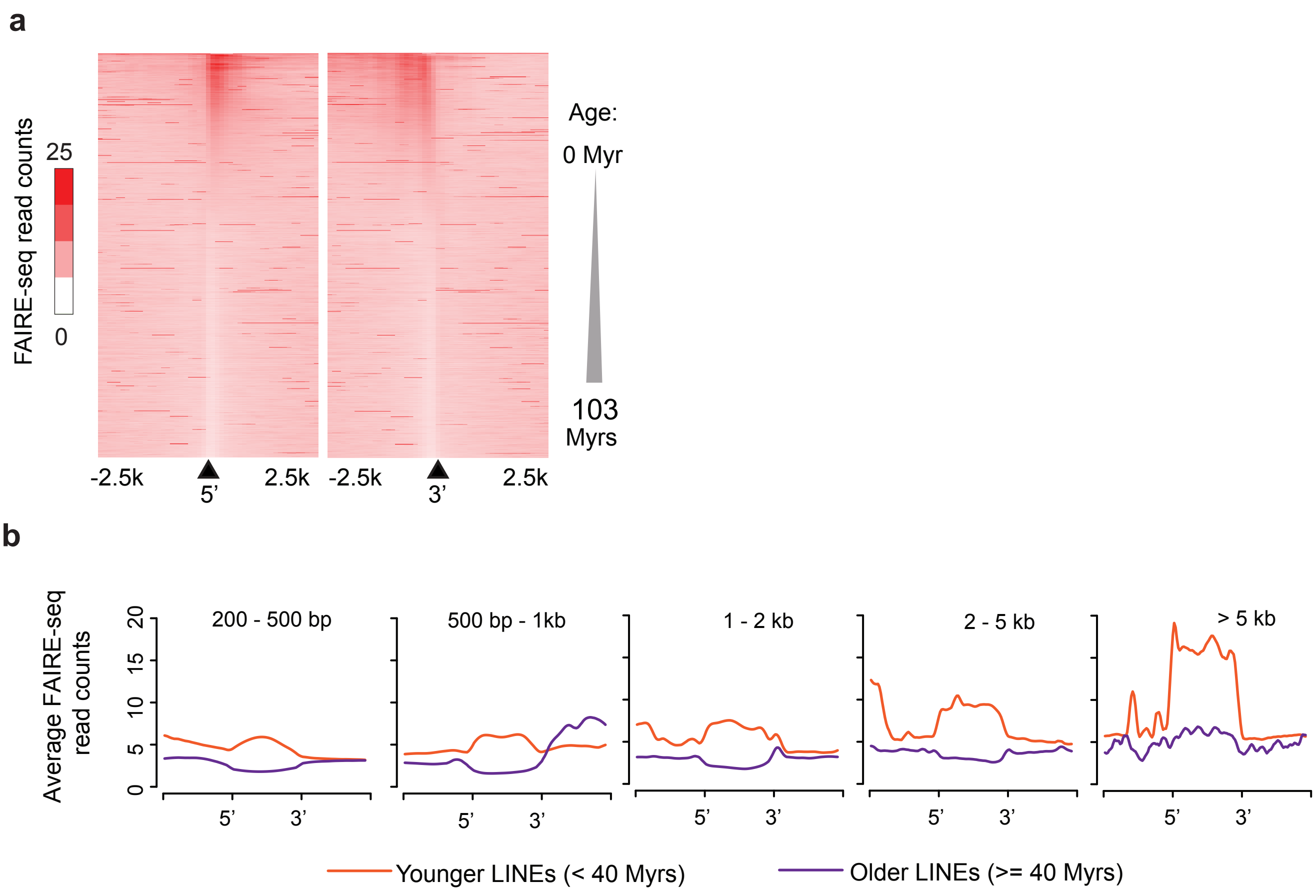

Figure S9. Differential chromatin accessibility profile at younger and older LINEs in A/J mice liver. (**a**) Heatmap showing FAIRE-seq read counts from A/J mice liver around 5' (left) and 3' (right) regions of LINEs. LINEs are sorted by their evolutionary age. Black triangles denote the 5' (left) or the 3' (right) end of LINEs, with plots extending +/- 2,500 bp upstream and downstream. (**b**) Aggregate plots of average FAIRE-seq read counts upstream, downstream and within LINEs, organized by size of LINEs.

Figure S10

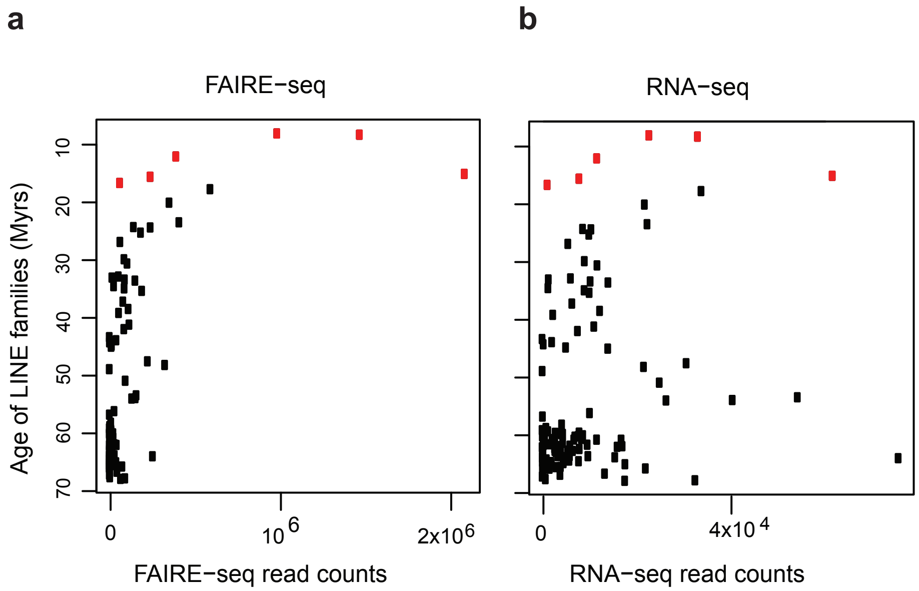

Figure S10: Accessibility and transcription of LINE subfamilies. (**a**,**b**) C57BL/6J liver FAIRE-seq (**a**) and RNA-seq (**b**) read counts of LINE families against the average age of LINEs in the family. Read counts for TE families are generated by using TEtranscripts (Supplementary Methods). L1Md families of LINEs are colored in red.

Figure S11

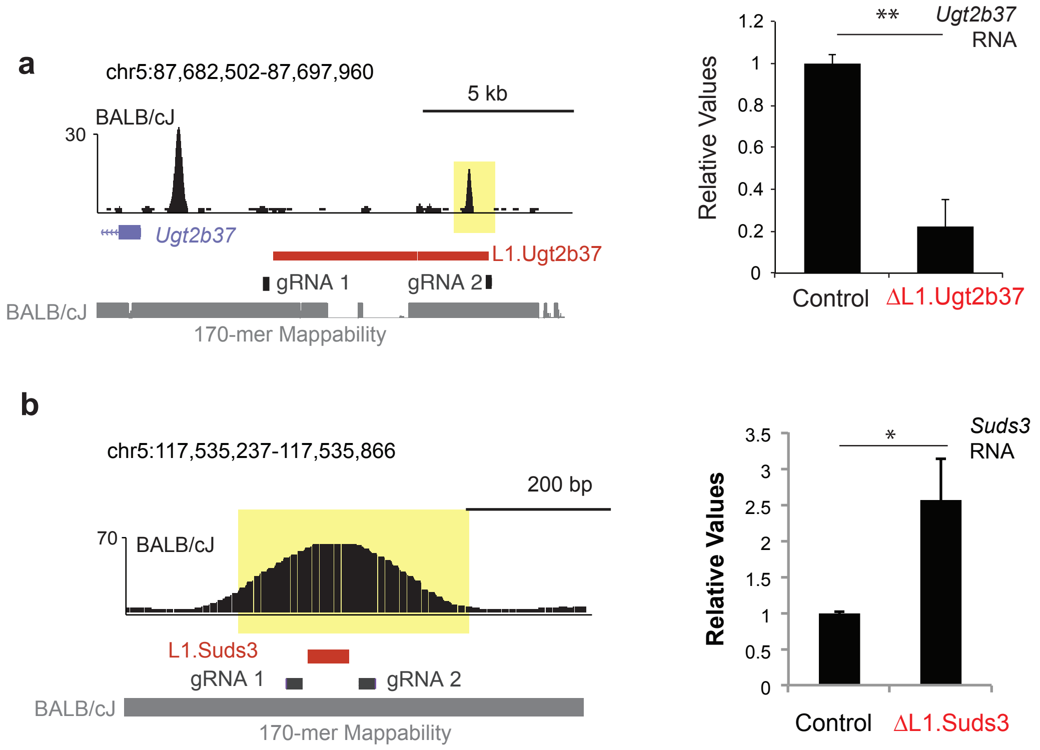

Figure S11. CRISPR-Cas9 deletion of additional TEs. (**a-b**) Genome browser view of deleted TE site. Shown are the BALB/cJ FAIRE-seq wiggle tracks, RefSeq genes, deleted TE, guide RNAs and 170-mer BALB/cJ pseudo-genome mappability tracks. Quantitative PCR of nearby genes expression level in H2.35 cells. Control H2.35 cells were transfected with empty vectors. ** $p < 0.01$, * $p < 0.05$, Student's t-test (n=3).

Figure S12

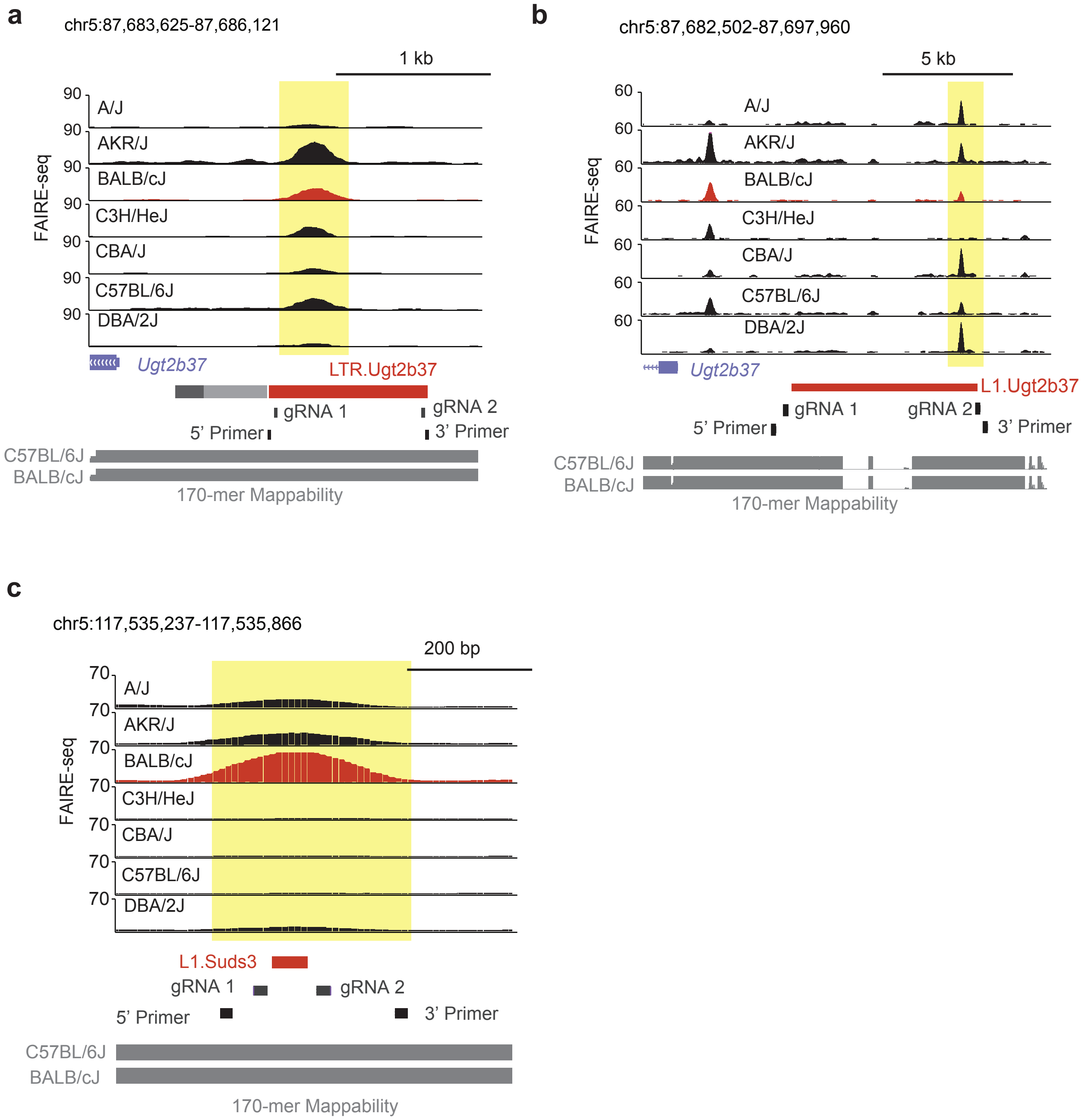

Figure S12. Guide RNA and genotyping primers used for CRISPR-Cas9 genome editing. (**a-c**) Genome browser views of guide RNAs and primers used for CRISPR-Cas9 genome editing. Shown are the seven FAIRE-seq wiggle tracks, RefSeq genes, RepeatMasker TEs, guide RNA and genotyping primer locations, and 170-mer mappability tracks of reference genome and a representative pseudo-genome.

Figure S13

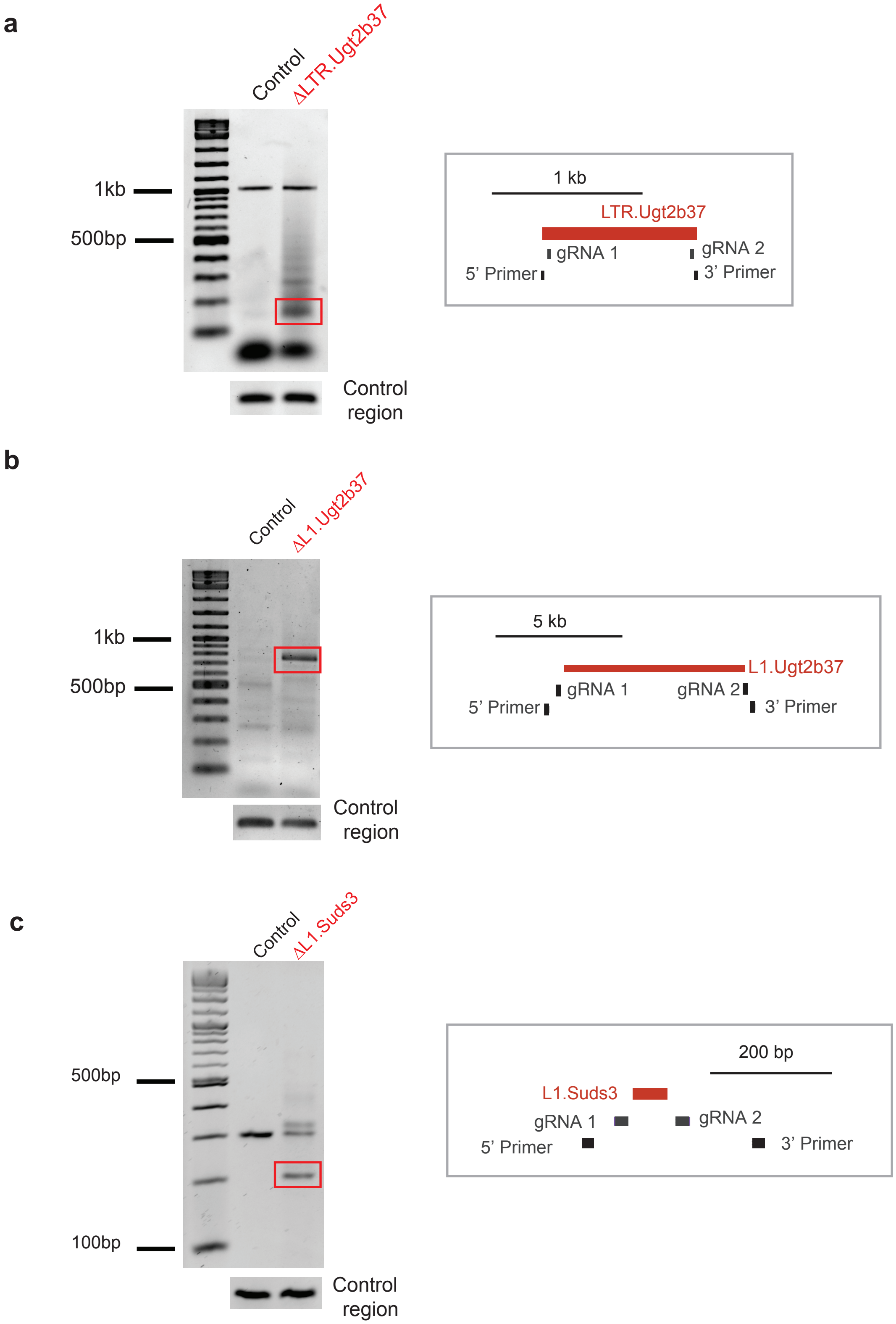

Figure S13. Genotyping for TE deletions. (**a-c**) PCR validation on control (cells transfected with empty vectors) or mutant genomic DNA, using primers flanking the expected deletion. Genotyping and control genomic region PCR primers are in Table S4.

Figure S14

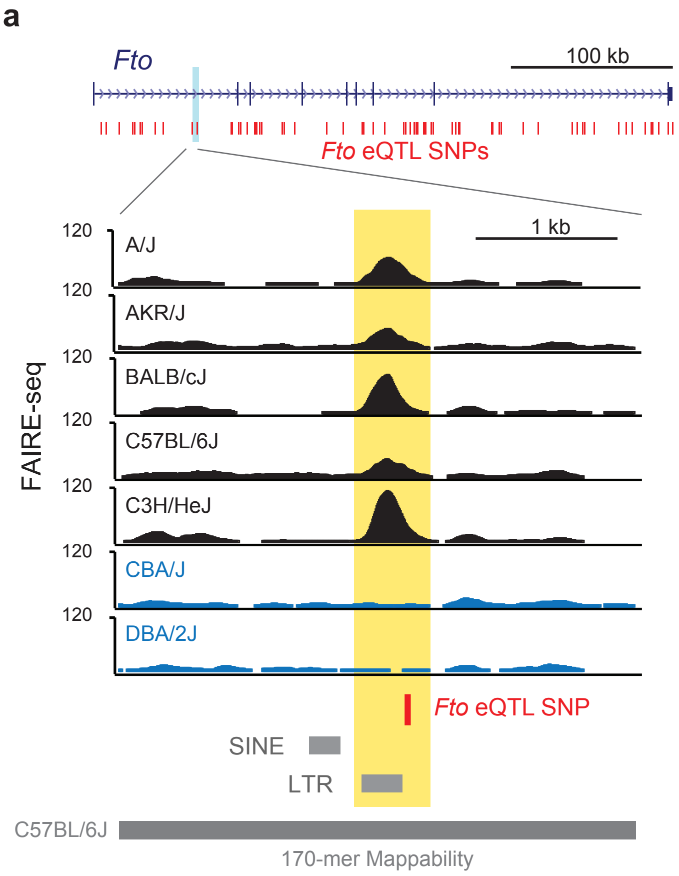

Figure S14. Example of an eQTL that associated with variable chromatin accessibility at an LTR. Shown are the seven FAIRE-seq wiggle tracks, RefSeq genes, RepeatMasker TEs, 170-mer mappability tracks, and eQTLs SNPs associated with *Fto* (Parks, *et al.* Cell Metab, 2013). Strains in black have the mm9 reference genotype, while the strains in blue have an alternative genotype.

Table S1. **Summary of FAIRE-seq data sets in all the strains in this study.** Listed are the numbers of reads mapped uniquely to strain-specific reference genome, reads filtered for pairing and PCR duplicates, and total number of reproducible FAIRE peaks identified in each strains.

Strain	Sample	Total read $x10^6$	Reads post filter $x10^6$	FAIRE peak identified by F-seq & IDR
A/J	Rep1	13,836,220	12,331,312	29,914
	Rep2	14,323,695	12,159,127	
AKR/J	Rep1	25,120,508	21,888,481	29,923
	Rep2	27,067,431	23,766,117	
BALB/cJ	Rep1	19,185,543	12,556,928	30,077
	Rep2	20,374,839	13,877,891	
C57BL/6J	Rep1	22,484,699	20,942,285	30,598
	Rep2	24,519,376	23,343,598	
C3H/HeJ	Rep1	20,400,719	12,830,230	30,370
	Rep2	25,350,052	18,635,238	
CBA/J	Rep1	23,497,233	20,541,717	30,613
	Rep2	22,170,236	18,426,726	
DBA/2J	Rep1	16,778,315	15,883,801	26,769
	Rep2	20,831,992	19,808,014	
BXH2/TyJ	Rep1	24,213,505	23160629	30,371
	Rep2	17,585,516	15,504,838	
BXH19/TyJ	Rep1	20,336,082	14,919,820	30,499
	Rep2	16,812,480	8,934,168	

Table S3. **Enriched biological process from GREAT analysis of accessible chromatin sites.** Genomic coordinates of accessible chromatin sites were used as input for Genomic Regions Enrichment of Annotations Tool (GREAT) analysis (see Materials and Methods).

Chromatin sites		Total enriched GO terms*	GO biological process	-log10 (*p*-value)
Variable	TE (934)	9	negative regulation of cellular carbohydrate metabolic process	5.29
			regulation of gluconeogenesis	4.86
			negative regulation of carbohydrate metabolic process	4.73
			negative regulation of insulin secretion involved in cellular response to glucose stimulus	4.64
			regulation of lipid storage	4.48
			regulation of glucose metabolic process	4.46
			negative regulation of gluconeogenesis	4.43
			negative regulation of lipid storage	4.37
			negative regulation of peptide hormone secretion	4.14
	Younger LINEs (164)	2	negative regulation of gluconeogenesis	7.98
			negative regulation of cellular carbohydrate metabolic process	7.93
	No repeats (1056)	2	filopodium assembly	4.98
			antigen processing and presentation of peptide antigen via MHC class I	4.29
	Other repeats (549)	0		
Common	TE (822)	5	cellular response to peptide hormone stimulus	4.94
			oxaloacetate metabolic process	4.13
			regulation of triglyceride metabolic process	4.01
			response to peptide hormone stimulus	3.99
			triglyceride metabolic process	3.97
	No repeats (1428)	7	cellular response to hydrogen peroxide	7.00
			cellular response to oxidative stress	6.08
			cellular response to reactive oxygen species	5.55
			intestinal absorption	5.38
			response to hydrogen peroxide	4.57
			unsaturated fatty acid biosynthetic process	3.98
			regulation of glycolysis	3.83
	Other repeats (289)	0		

Table S4. **Sequences used in this study.**

S3-1: gRNA sequences

	**Left gRNA + PAM**	**Right gRNA + PAM**
LTR.Ugt2b37	CAATTGGTTTATCAACTCGC + AGG	GGGACTCATCGAAGTTTTTG + GGG
L1.Ugt2b37	TTTTACTCTTAGCAGTCTGT + TGG	AATTTCTGTAATTGGGCCCC + AGG
L1.Suds3	TCCTCTGGGGACATGACCGC + TGG	TGTGGCTCTTGGAATGGTAG + GGG

S3-2: Genotyping primers

	**Flanking fwd**	**Flanking Rev**
LTR.Ugt2b37	GGGAAATGCTATACACACACCA	CATTATGTGCTGTGGACTGGA
L1.Ugt2b37	CCAACTATCCCATGGCTCCT	GCTGGGCTTCAACAACATCA
L1.Suds3	ACAGAAGACCCGAGAACACC	GATCATGTCCACCTCCCTCC

S3-3:qPCR primers

	**Fwd primer**	**Rev primer**
Ugt2b37	GCCGATGGAATTCAGTCATT	GACTTCATGGCCCCTCTGTA
Suds3	CCTTGTTCAGGGTGGAAGAA	CAGTAGGGCACGTGATGAGA
Genotyping control region	GCGTGTCGGGGCCAAATCCA	TTTCTAGCCCCAGCTGACGCGC
STAT3-ChIP qPCR (L1Md_F2)	GGACACTTTGCCCCTTCTTA	TTATGGGATGGATCTCTGCAT
Bisulfite Sanger sequencing (Fig. 7c)	AGGGGTTTTAGGTTTTAGGAAGAG	CCAAAACTACTCAAAAAAACCCTATC
